# Supplementary material for: Land Use Influences the Composition and Antimicrobial Effects of Propolis
Source: Insects. 2022 Feb 28;13(3):239. doi: 10.3390/insects13030239 (PMC8950720; doi:10.3390/insects13030239)
Supplement: Supplementary file 1 [file insects-13-00239-s001.zip › insects-1593358-supplementary-File S1.pdf]

Acq On : 7 Mar 2019 00:57
Operator :
Sample : Apiary B 25 :
Misc
ALS Vial : 6 Sample Multiplier: 1

Integration Parameters: autoint1.e
Integrator: ChemStation

peak #	R.T. min	first scan	max scan	last scan	PK TY	peak height	corr. area	corr. % max.	% of total
1	4.479	208	243	273	BB 4	25407	1151311	0.33%	0.197%
2	6.099	466	527	537	BV 2	19354	136098	0.04%	0.023%
3	6.869	597	661	697	PB 3	12985	2606699	0.75%	0.445%
4	8.295	891	910	1040	PB 2	302619	25250128	7.22%	4.310%
5	9.449	1083	1112	1151	BB 3	42391	2569839	0.73%	0.439%
6	11.183	1385	1415	1458	BB 2	24539	1770732	0.51%	0.302%
7	14.525	1739	1999	2149	BV 2	825650	349872270	100.00%	59.720%
8	15.522	2149	2173	2373	VB 3	781947	79704452	22.78%	13.605%
9	19.464	2776	2862	2922	BV	388966	36997953	10.57%	6.315%
10	19.831	2922	2926	2935	VV	24902	1067588	0.31%	0.182%
11	19.906	2935	2940	2981	VB	24148	1879256	0.54%	0.321%
12	23.802	3447	3620	3696	PB 9	29107	8926748	2.55%	1.524%
13	25.228	3783	3869	3941	BB 9	31369	4697798	1.34%	0.802%
14	26.318	3945	4060	4070	BV 9	8708	-1693442	-0.48%	-0.289%
15	26.445	4070	4082	4095	VB 9	13020	624715	0.18%	0.107%
16	27.115	4113	4199	4255	BV 9	17369	4728674	1.35%	0.807%
17	27.513	4255	4269	4284	VV 9	10665	630726	0.18%	0.108%
18	33.704	5263	5351	5476	BV 9	39540	10735875	3.07%	1.833%
19	35.047	5516	5586	5754	BV 9	56650	14538052	4.16%	2.482%
20	38.554	6064	6198	6433	BV 4	97034	34030920	9.73%	5.809%
21	43.132	6856	6999	7091	PB 4	14910	5628959	1.61%	0.961%

Sum of corrected areas: 585855350

Acq On : 7 Mar 2019 2:27
Operator :
Sample : Apiary B 32 :
Misc
ALS Vial : 7 Sample Multiplier: 1

Integration Parameters: autoint1.e
Integrator: ChemStation

peak #	R.T. min	first scan	max scan	last scan	PK TY	peak height	corr. area	corr. % max.	% of total
1	4.476	208	243	275	BB 4	18943	857757	0.45%	0.202%
2	6.101	498	527	535	BV 2	16625	240022	0.13%	0.057%
3	7.026	598	688	714	PV	15897	4225991	2.22%	0.995%
4	7.247	714	727	737	VV 5	11642	517715	0.27%	0.122%
5	8.297	890	911	961	PV 2	224343	14524101	7.65%	3.421%
6	9.452	1084	1112	1185	BV 2	50797	4181618	2.20%	0.985%
7	11.183	1386	1415	1473	BB 3	25889	2031990	1.07%	0.479%
8	14.239	1741	1949	2053	BV 3	538204	189970011	100.00%	44.746%
9	14.886	2053	2062	2099	VB 9	22485	1673653	0.88%	0.394%
10	15.519	2134	2173	2327	BB 3	443630	43005587	22.64%	10.130%
11	19.468	2748	2863	2981	BV 2	301504	35895026	18.90%	8.455%
12	20.209	2981	2992	3032	VB 6	12015	1238921	0.65%	0.292%
13	23.807	3540	3621	3728	BV 6	27994	10119746	5.33%	2.384%
14	25.251	3837	3873	3950	BV 8	24428	3772744	1.99%	0.889%
15	26.310	3950	4059	4069	PV 8	30643	2838453	1.49%	0.669%
16	26.456	4069	4084	4100	VV 8	38467	3026341	1.59%	0.713%
17	27.159	4100	4207	4232	VV 8	44909	16140824	8.50%	3.802%
18	27.527	4232	4271	4278	VV 8	50626	7231413	3.81%	1.703%
19	27.640	4278	4291	4352	VV 8	56159	11149293	5.87%	2.626%
20	28.074	4352	4367	4495	VB 8	39224	8446450	4.45%	1.990%
21	33.764	5268	5361	5461	BV 8	34842	8264945	4.35%	1.947%
22	35.138	5503	5601	5861	BV 8	133991	37784165	19.89%	8.900%
23	38.686	6086	6222	6383	BV 6	45058	17414022	9.17%	4.102%

Sum of corrected areas: 424550786

File
Operator :
Acquired : 7 Mar 2019 2:27 using AcqMethod FOMETHOD.M
Instrument : 5975 MSD
Sample Name: Apiary B 32
Misc Info :
Vial Number: 7

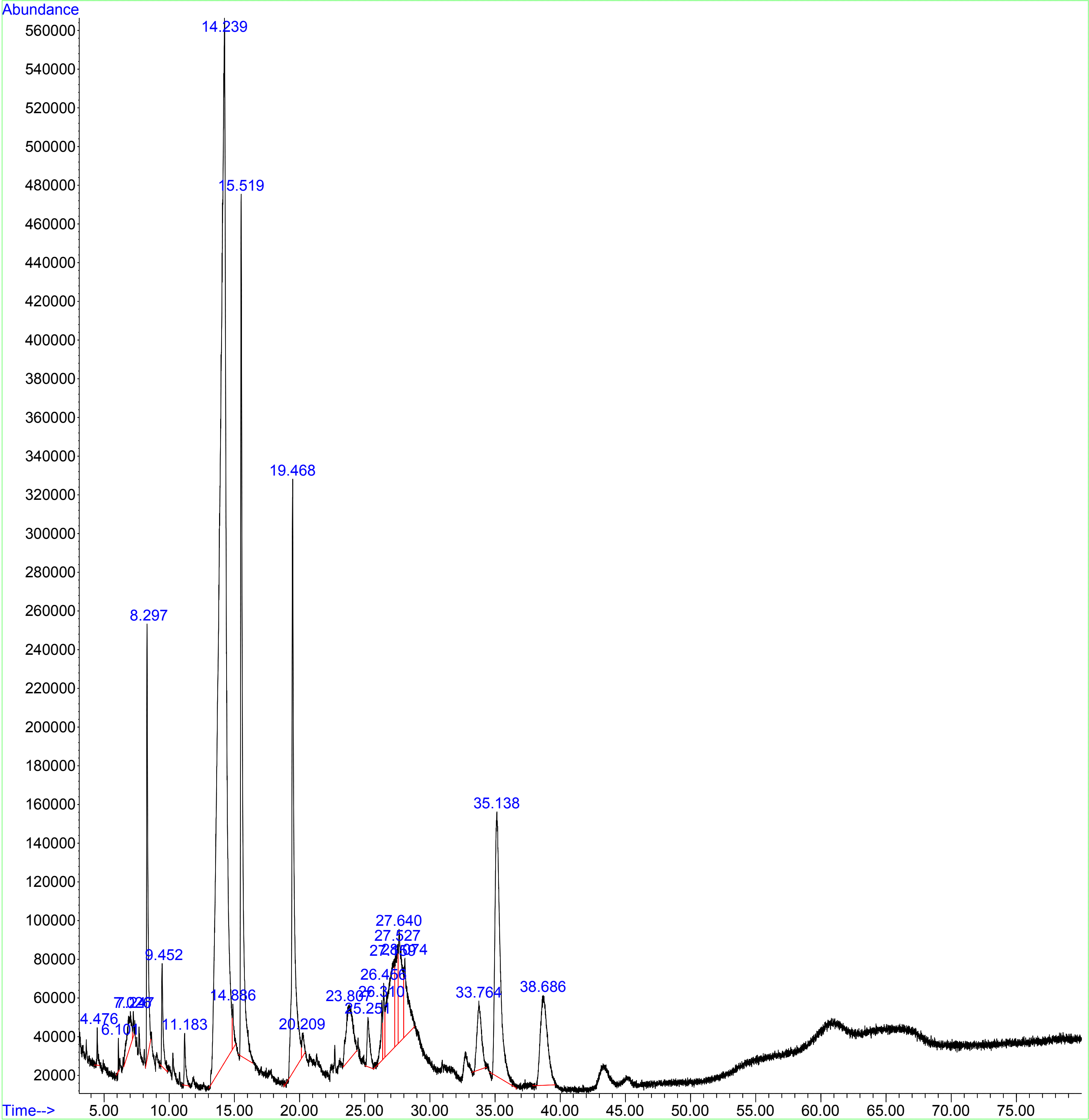

Acq On : 7 Mar 2019 3:56
Operator :
Sample : Apiary B 33 :
Misc
ALS Vial : 8 Sample Multiplier: 1

Integration Parameters: autoint1.e
Integrator: ChemStation

peak #	R.T. min	first scan	max scan	last scan	PK TY	peak height	corr. area	corr. % max.	% of total
1	3.640	71	97	116	BB	33992	807262	0.09%	0.042%
2	4.490	215	245	303	BB 3	34526	3000173	0.34%	0.157%
3	6.101	502	527	537	BV 2	27081	563270	0.06%	0.029%
4	6.611	601	616	638	VV 2	19433	1525844	0.17%	0.080%
5	6.797	638	648	670	VV 9	22713	1318552	0.15%	0.069%
6	7.258	670	729	740	VV 4	23611	2784848	0.31%	0.145%
7	7.360	740	747	779	VB 5	16393	1079382	0.12%	0.056%
8	8.299	887	911	1018	PV 2	535163	45624999	5.14%	2.381%
9	9.456	1082	1113	1165	BV 3	102757	7082054	0.80%	0.370%
10	10.296	1197	1260	1284	PV 3	32500	2675055	0.30%	0.140%
11	10.464	1284	1289	1306	VV 10	14195	645379	0.07%	0.034%
12	11.187	1386	1416	1468	BB 3	19315	1562510	0.18%	0.082%
13	13.201	1739	1768	1781	BV 3	37702	1689385	0.19%	0.088%
14	15.363	1781	2145	2164	VV 3	1658331	888370074	100.00%	46.368%
15	15.545	2164	2177	2429	VV 2	1783827	240867170	27.11%	12.572%
16	19.490	2800	2867	2985	BV 2	834637	99466498	11.20%	5.192%
17	20.280	2985	3005	3082	VB 5	50630	9024202	1.02%	0.471%
18	23.911	3449	3639	3644	BV 8	61829	11953588	1.35%	0.624%
19	23.984	3644	3652	3727	VV 8	60007	11907105	1.34%	0.621%
20	24.494	3727	3741	3796	VB 6	74274	4766078	0.54%	0.249%
21	25.278	3842	3878	3892	BV 7	61652	5546158	0.62%	0.289%
22	25.417	3892	3902	3950	VV 8	63941	5069671	0.57%	0.265%
23	26.315	3982	4059	4072	PV 8	142134	9048311	1.02%	0.472%
24	26.462	4072	4085	4112	VV 4	188726	14498063	1.63%	0.757%
25	26.931	4112	4167	4189	VV 4	86055	19598343	2.21%	1.023%
26	27.168	4189	4208	4217	VV 4	91476	8250790	0.93%	0.431%
27	27.541	4217	4274	4285	VV 6	204421	30628280	3.45%	1.599%
28	27.660	4285	4295	4307	VV 9	223804	15538377	1.75%	0.811%
29	27.803	4307	4320	4325	VV 9	201828	12229206	1.38%	0.638%
30	28.096	4325	4371	4387	VV 9	282885	48671772	5.48%	2.540%
31	28.449	4387	4432	4450	VV 10	290388	59677503	6.72%	3.115%
32	28.623	4450	4463	4496	VV 10	315665	47857581	5.39%	2.498%
33	28.891	4496	4510	4538	VV 10	306267	41880126	4.71%	2.186%
34	29.086	4538	4544	4553	VV 8	291812	14344862	1.61%	0.749%
35	29.164	4553	4557	4853	VV 3	293549	107046513	12.05%	5.587%
36	33.823	5294	5372	5467	BV 3	39500	9436066	1.06%	0.493%
37	35.222	5530	5616	5868	BB 3	234885	72350527	8.14%	3.776%
38	38.834	6101	6247	6491	BB 4	118828	53563270	6.03%	2.796%
39	43.463	6915	7056	7132	BB 6	14421	4505744	0.51%	0.235%
40	64.531	10489	10738	10743	BV 6	8076	-2284656	-0.26%	-0.119%
41	64.917	10743	10806	10837	VB 6	9034	1747049	0.20%	0.091%

Sum of corrected areas: 1915916985

Acq On : 7 Mar 2019 5:26
Operator :
Sample : Apiary B 39 :
Misc
ALS Vial : 9 Sample Multiplier: 1

Integration Parameters: autoint1.e
Integrator: ChemStation

peak #	R.T. min	first scan	max scan	last scan	PK TY	peak height	corr. area	corr. % max.	% of total
1	3.640	42	97	115	BB	36238	1142367	0.07%	0.036%
2	4.482	215	244	268	BV 3	18656	838685	0.05%	0.026%
3	6.101	501	527	539	BV 3	31712	752871	0.04%	0.024%
4	6.492	572	595	603	BV	19034	591155	0.03%	0.019%
5	6.606	603	615	639	VV 9	26257	1929388	0.11%	0.060%
6	6.796	639	648	671	VB 4	51541	1640896	0.10%	0.051%
7	8.303	890	912	1094	PB	647610	55798869	3.29%	1.747%
8	9.451	1095	1112	1162	BV	147297	8394520	0.50%	0.263%
9	10.305	1224	1262	1308	BV 3	43373	4106255	0.24%	0.129%
10	11.187	1386	1416	1480	BB 3	57033	4154003	0.25%	0.130%
11	13.205	1742	1768	1781	BV 7	20820	883065	0.05%	0.028%
12	15.579	1781	2183	2216	VV 3	4095955	1694492313	100.00%	53.057%
13	15.876	2216	2235	2544	VV	3397204	708238077	41.80%	22.176%
14	19.515	2781	2871	2985	BV 2	1672405	203025167	11.98%	6.357%
15	20.319	2985	3012	3083	VV 5	121139	27367802	1.62%	0.857%
16	20.877	3083	3109	3166	VV 5	38826	8013581	0.47%	0.251%
17	21.338	3166	3190	3257	VV 5	49096	6364121	0.38%	0.199%
18	23.660	3529	3595	3605	BV 5	38590	3359179	0.20%	0.105%
19	24.093	3605	3671	3727	VV 5	66841	22835543	1.35%	0.715%
20	24.499	3727	3742	3802	VV 8	128204	11202066	0.66%	0.351%
21	25.308	3842	3883	3895	VV 5	160100	14575342	0.86%	0.456%
22	25.412	3895	3902	3953	VV 8	146309	14151277	0.84%	0.443%
23	25.788	3953	3967	4000	VV 8	39165	2328135	0.14%	0.073%
24	26.321	4043	4061	4073	VV 3	214117	9451325	0.56%	0.296%
25	26.468	4073	4086	4117	VV 5	227667	13552229	0.80%	0.424%
26	26.913	4117	4164	4182	VV 5	47253	6913799	0.41%	0.216%
27	27.154	4182	4206	4225	VV 5	48000	5150925	0.30%	0.161%
28	27.551	4248	4276	4287	VV 5	261816	14778215	0.87%	0.463%
29	27.672	4287	4297	4337	VV 5	147477	14523101	0.86%	0.455%
30	28.093	4337	4370	4394	VV 9	192657	20294547	1.20%	0.635%
31	28.646	4394	4467	4493	VV 9	125047	36201272	2.14%	1.134%
32	28.920	4493	4515	4526	VV 9	124874	13676097	0.81%	0.428%
33	29.275	4526	4577	4582	VV 9	174362	27325026	1.61%	0.856%
34	29.335	4582	4587	4610	VV 9	181845	16069867	0.95%	0.503%
35	29.504	4610	4617	4791	VV 6	161077	47081552	2.78%	1.474%
36	33.883	5301	5382	5556	BV 6	44261	15878256	0.94%	0.497%
37	35.299	5556	5630	5868	PV 6	101882	33858838	2.00%	1.060%
38	39.006	6139	6277	6567	BV 4	222216	116887029	6.90%	3.660%
39	43.839	6974	7122	7147	BV 7	13203	5881087	0.35%	0.184%

Sum of corrected areas: 3193707839

File
Operator :
Acquired : 7 Mar 2019 5:26 using AcqMethod FOMETHOD.M
Instrument : 5975 MSD
Sample Name: Apiary B 39
Misc Info :
Vial Number: 9

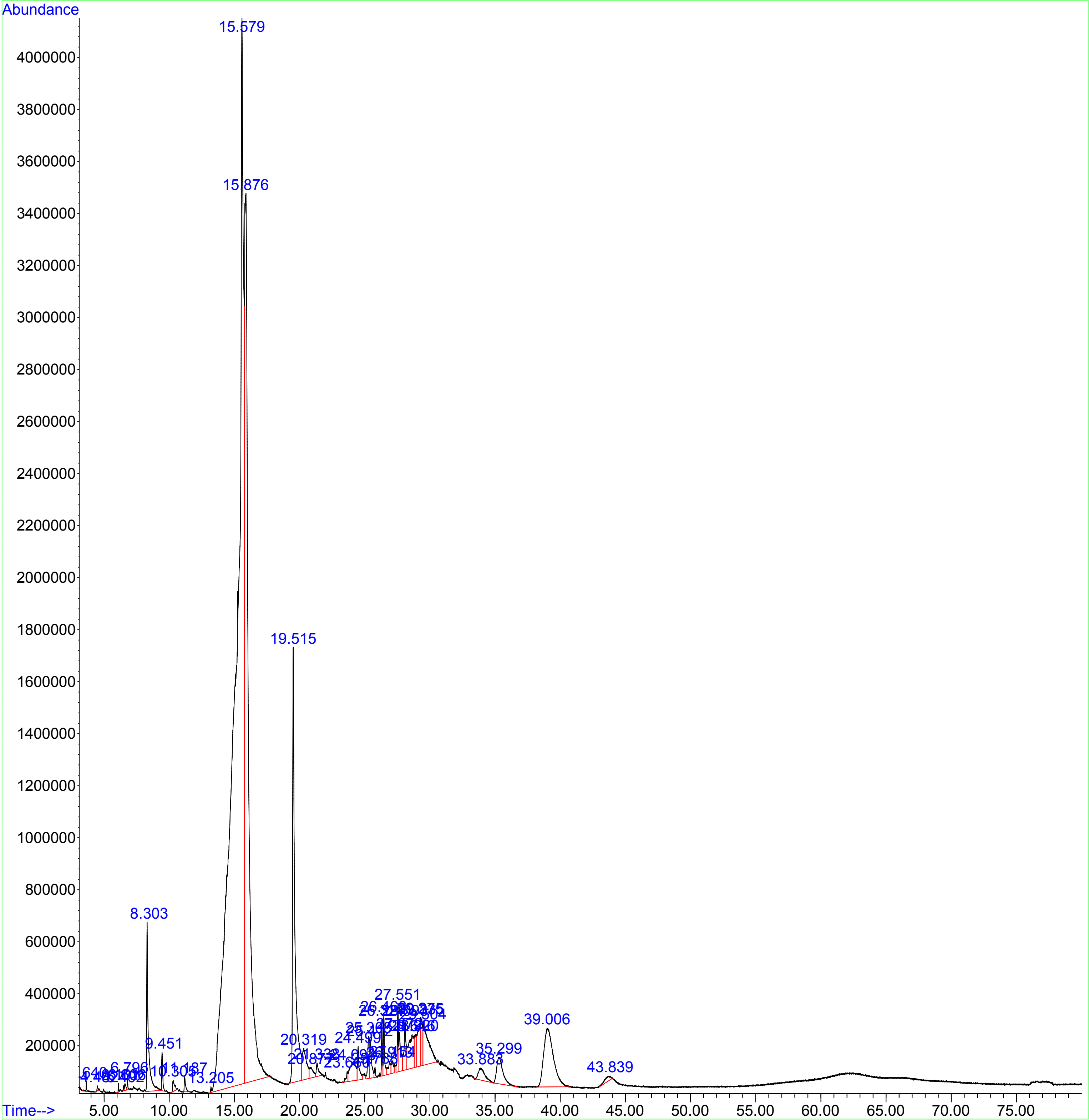

Acq On : 7 Mar 2019 6:55
Operator :
Sample : Apiary B 41 :
Misc
ALS Vial : 10 Sample Multiplier: 1

Integration Parameters: autoint1.e
Integrator: ChemStation

peak #	R.T. min	first scan	max scan	last scan	PK TY	peak height	corr. area	corr. % max.	% of total
1	3.641	71	97	118	BB	35212	885365	0.13%	0.047%
2	4.487	185	245	268	BV 3	32197	747910	0.11%	0.039%
3	6.102	500	527	539	BV 2	29548	682286	0.10%	0.036%
4	6.798	640	649	664	VV 10	18415	597277	0.09%	0.031%
5	8.306	889	912	1072	PB	575835	49389075	7.21%	2.602%
6	9.456	1083	1113	1166	BV 4	102031	6815033	1.00%	0.359%
7	10.307	1227	1262	1285	BV 4	27853	2215541	0.32%	0.117%
8	10.470	1285	1290	1310	VV 4	16243	784641	0.11%	0.041%
9	11.201	1386	1418	1500	BB 3	54928	5096882	0.74%	0.268%
10	13.210	1705	1769	1779	BV 3	27308	390523	0.06%	0.021%
11	14.379	1779	1973	1979	VV	600672	176268499	25.74%	9.285%
12	15.392	1979	2151	2167	VV 3	1608646	684824960	100.00%	36.075%
13	15.569	2167	2182	2423	VV 2	2123413	337344779	49.26%	17.771%
14	19.506	2792	2870	2990	BV 2	811701	113660308	16.60%	5.987%
15	20.328	2990	3013	3079	VV 6	61837	13331408	1.95%	0.702%
16	21.345	3175	3191	3254	VV 6	24444	2684358	0.39%	0.141%
17	23.495	3530	3567	3573	BV 6	27269	1632896	0.24%	0.086%
18	24.185	3573	3687	3728	VV 7	84120	31945483	4.66%	1.683%
19	24.498	3728	3742	3787	VV 7	88092	12407277	1.81%	0.654%
20	24.779	3787	3791	3810	VV 7	51581	3998736	0.58%	0.211%
21	24.925	3810	3817	3854	VV 7	49522	6798016	0.99%	0.358%
22	25.334	3854	3888	3928	VV 10	118328	21493471	3.14%	1.132%
23	25.593	3928	3933	3959	VV 10	56761	5004110	0.73%	0.264%
24	26.322	4047	4061	4072	VV 5	47506	2156840	0.31%	0.114%
25	26.471	4072	4087	4110	VV 4	75313	4610119	0.67%	0.243%
26	26.916	4120	4164	4183	VV 4	31491	5042135	0.74%	0.266%
27	27.153	4183	4206	4223	VV 4	35796	4175854	0.61%	0.220%
28	27.551	4258	4276	4285	VV 10	73290	4648062	0.68%	0.245%
29	27.682	4285	4298	4325	VV 10	80395	7559940	1.10%	0.398%
30	28.094	4325	4370	4390	VV 10	111237	14248614	2.08%	0.751%
31	28.656	4390	4469	4473	VV 6	121652	26130930	3.82%	1.377%
32	29.553	4473	4625	4630	VV 9	189377	86170854	12.58%	4.539%
33	29.607	4630	4635	5165	VV 8	185425	152774268	22.31%	8.048%
34	33.992	5305	5401	5560	BV 8	37259	13440044	1.96%	0.708%
35	35.378	5560	5643	5928	PB 8	77390	28474467	4.16%	1.500%
36	39.138	6156	6300	6661	BB 4	117481	69900375	10.21%	3.682%

Sum of corrected areas: 1898331337

File
Operator :
Acquired : 7 Mar 2019 6:55 using AcqMethod FOMETHOD.M
Instrument : 5975 MSD
Sample Name: Apiary B 41
Misc Info :
Vial Number: 10

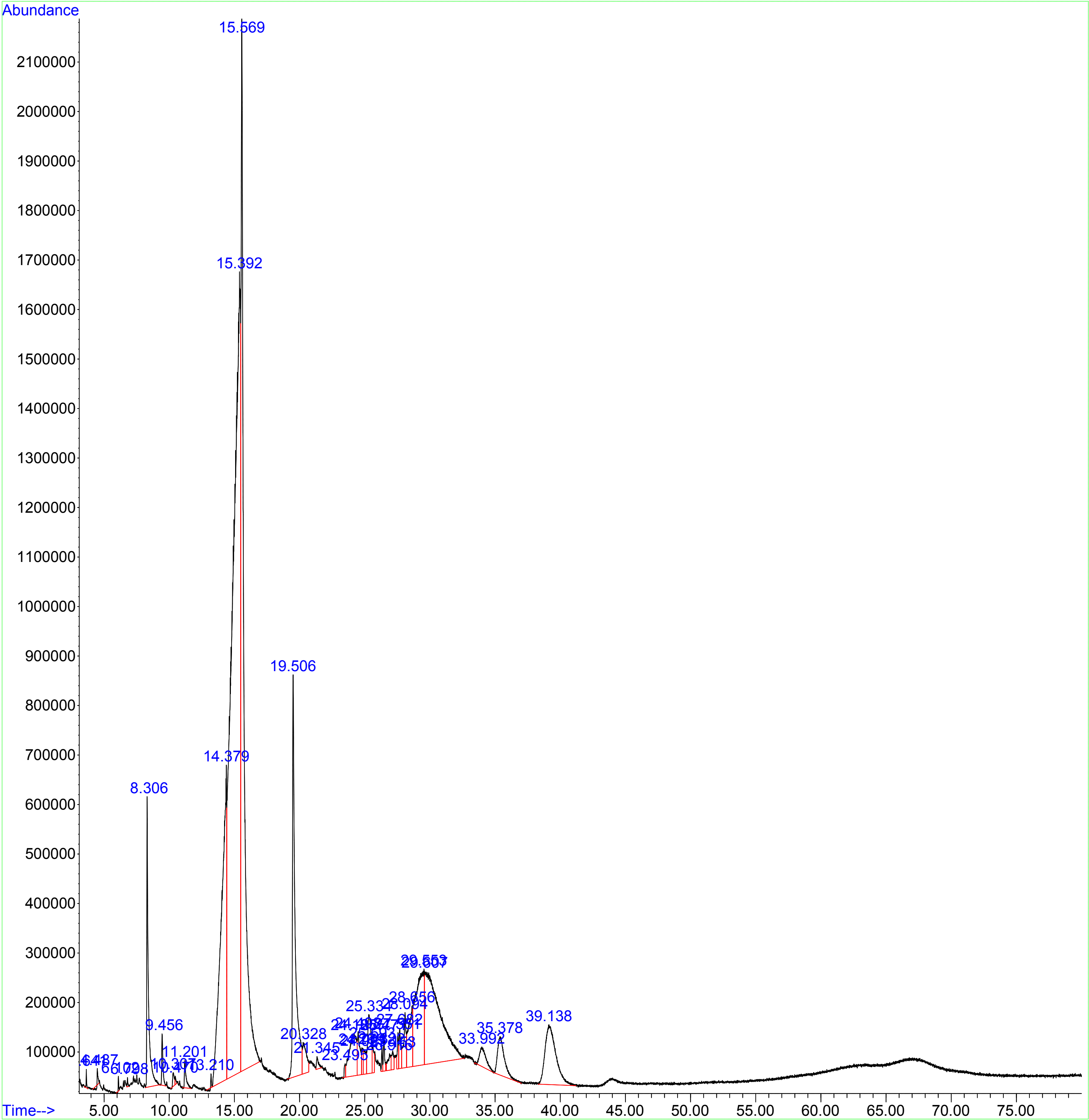

File
Operator :
Acquired : 7 Mar 2019 00:57 using AcqMethod FOMETHOD.M
Instrument : 5975 MSD
Sample Name: Apiary B 25
Misc Info :
Vial Number: 6

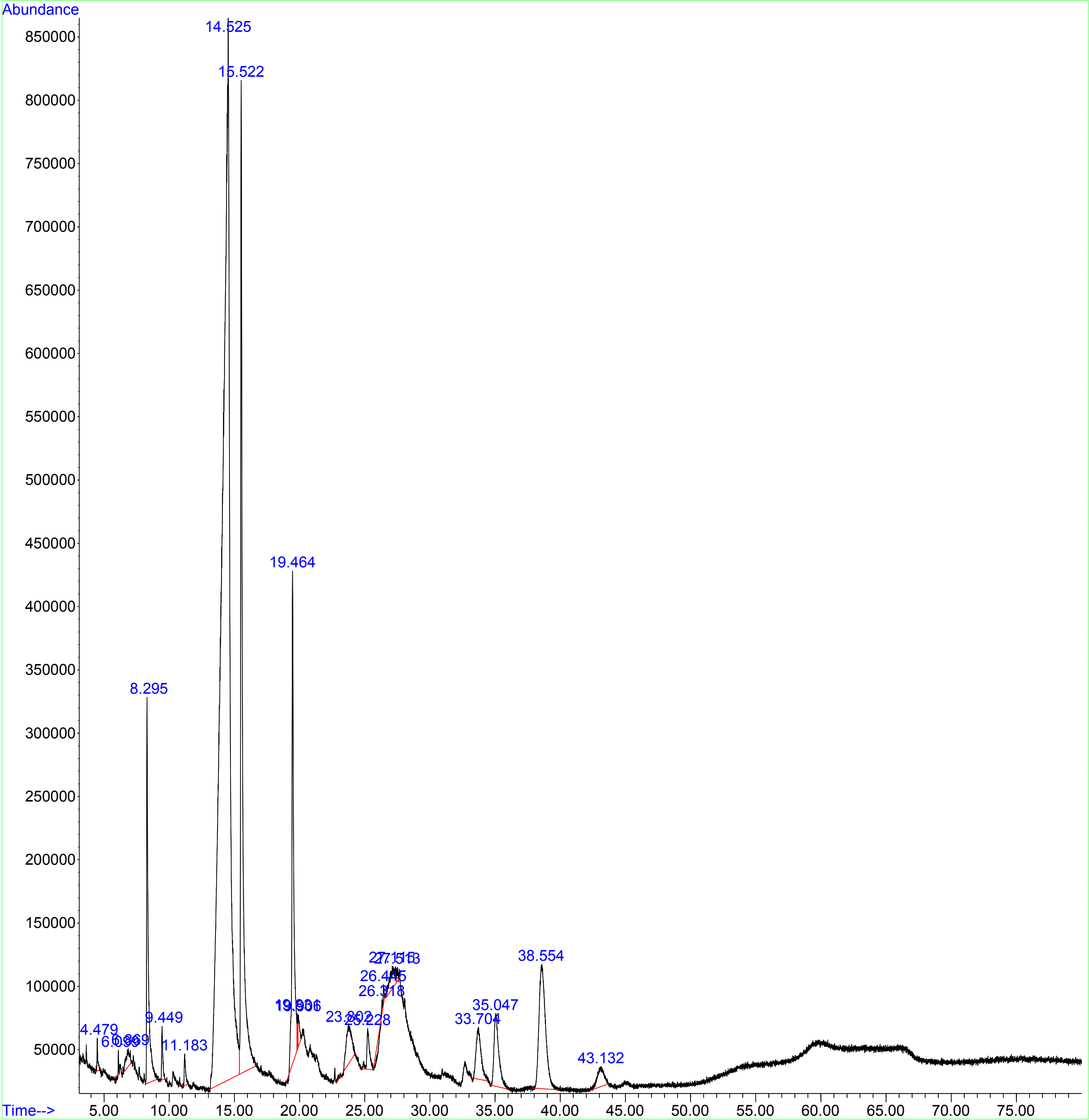

File
Operator :
Acquired : 7 Mar 2019 3:56 using AcqMethod FOMETHOD.M
Instrument : 5975 MSD
Sample Name: Apiary B 33
Misc Info :
Vial Number: 8

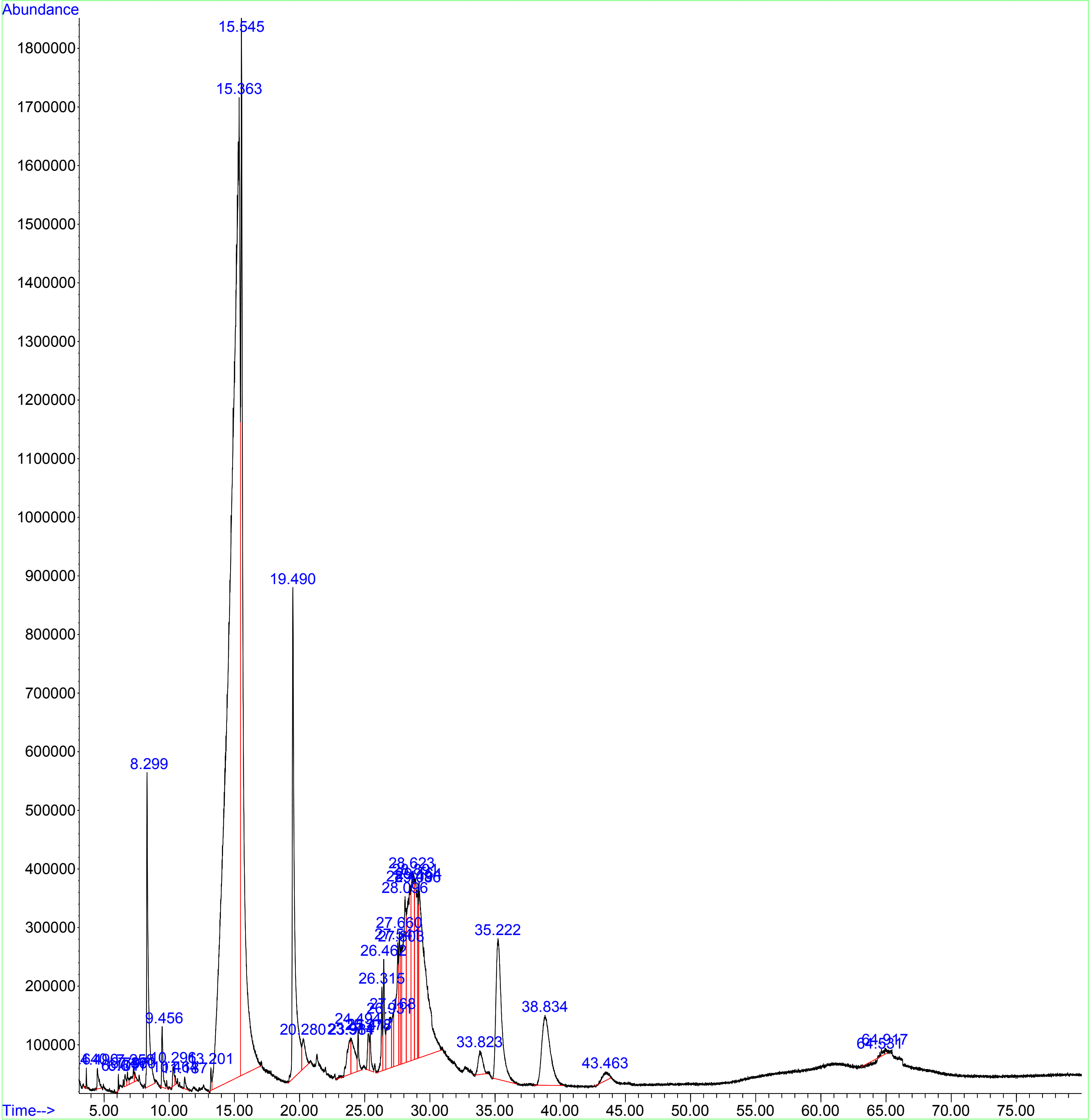

Acq On : 7 Mar 2019 8:25
Operator :
Sample : Apiary A 5
Misc :
ALS Vial : 11 Sample Multiplier: 1

Integration Parameters: autoint1.e
Integrator: ChemStation

peak #	R.T. min	first scan	max scan	last scan	PK TY	peak height	corr. area	corr. % max.	% of total
1	3.641	86	97	123	BB	32645	817306	0.14%	0.083%
2	4.485	217	244	268	BV 5	21072	884475	0.15%	0.090%
3	6.105	477	527	542	BV 4	20347	336886	0.06%	0.034%
4	8.312	887	913	1046	PV 2	512628	43326070	7.47%	4.419%
5	9.465	1086	1115	1162	BV 2	82932	5159757	0.89%	0.526%
6	10.329	1219	1266	1283	BV 4	30095	2276571	0.39%	0.232%
7	10.464	1283	1289	1311	VV 4	15992	816163	0.14%	0.083%
8	11.208	1387	1419	1475	BB 3	33313	2976856	0.51%	0.304%
9	15.037	1785	2088	2162	VV 3	1084700	579925747	100.00%	59.151%
10	15.585	2162	2184	2435	VV 3	1139711	171113712	29.51%	17.453%
11	19.512	2797	2871	2941	BV 2	568297	71393854	12.31%	7.282%
12	19.945	2941	2946	2994	VV 8	92204	12199678	2.10%	1.244%
13	20.291	2994	3007	3075	VV 5	54193	9486326	1.64%	0.968%
14	21.372	3160	3196	3227	BV 5	15992	1025660	0.18%	0.105%
15	24.506	3536	3743	3802	BV 8	34748	15863103	2.74%	1.618%
16	25.364	3802	3893	3960	VV 8	46687	10773395	1.86%	1.099%
17	26.324	4014	4061	4074	BV 8	28309	849728	0.15%	0.087%
18	26.473	4074	4087	4110	PV 6	28695	1443605	0.25%	0.147%
19	27.558	4110	4277	4287	PV 6	27906	5552808	0.96%	0.566%
20	27.678	4287	4298	4335	VB 6	20829	1689564	0.29%	0.172%
21	29.356	4441	4591	4640	BB 6	12269	3845991	0.66%	0.392%
22	34.123	5340	5424	5531	BB 6	21436	7318960	1.26%	0.747%
23	35.517	5563	5668	5829	BB 6	36818	12948402	2.23%	1.321%
24	39.241	6168	6318	6539	BB 4	32917	18391047	3.17%	1.876%

Sum of corrected areas: 980415665

File
Operator :
Acquired : 7 Mar 2019 8:25 using AcqMethod FOMETHOD.M
Instrument : 5975 MSD
Sample Name: Apiary A 5
Misc Info :
Vial Number: 11

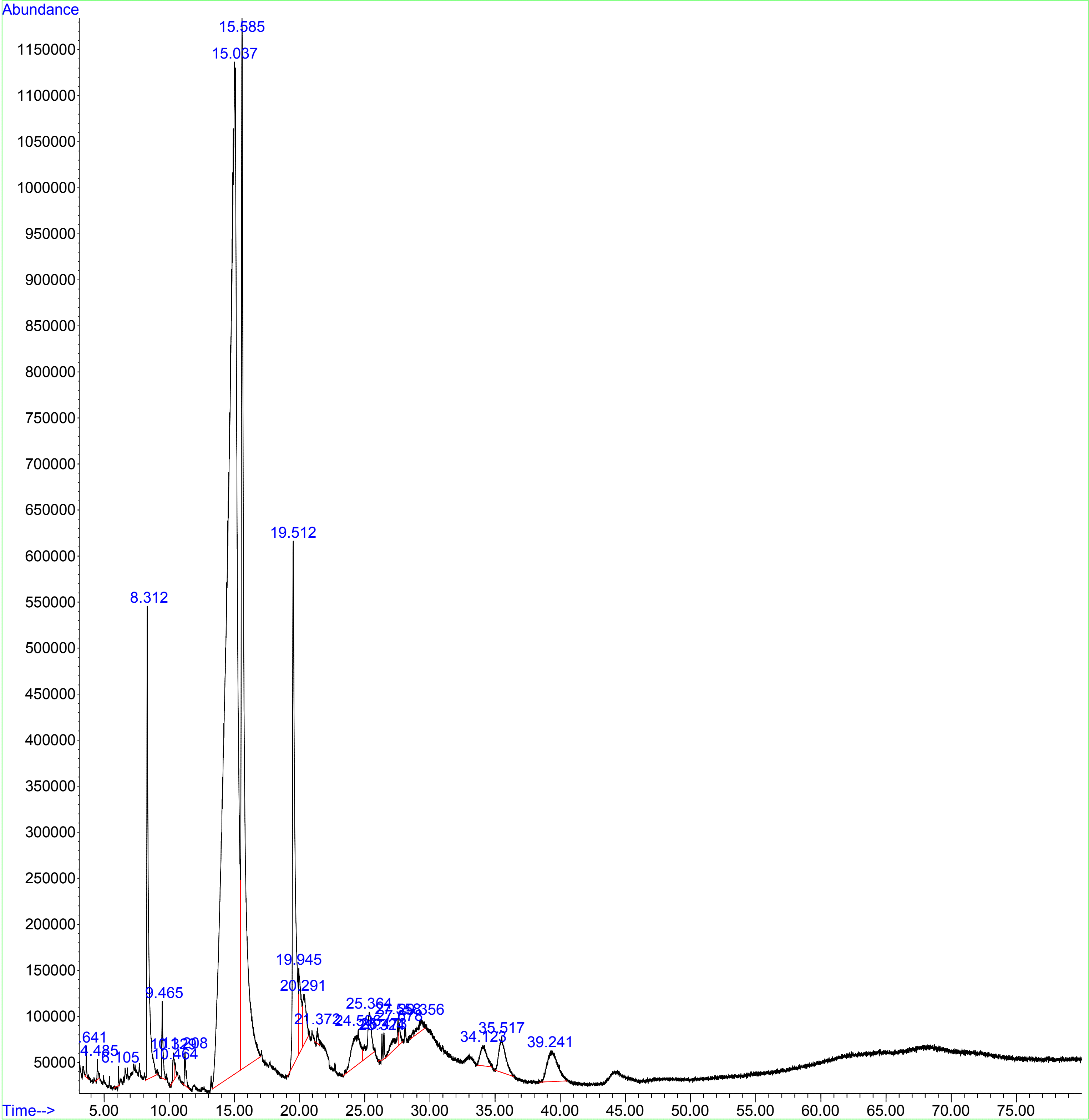

Acq On : 7 Mar 2019 9:54
Operator :
Sample : Apiary A 6
Misc :
ALS Vial : 12 Sample Multiplier: 1

Integration Parameters: autoint1.e
Integrator: ChemStation

peak #	R.T. min	first scan	max scan	last scan	PK TY	peak height	corr. area	corr. % max.	% of total
1	3.642	78	97	120	BB	19931	645635	0.15%	0.083%
2	4.480	219	243	268	BV 2	29710	1029139	0.23%	0.132%
3	6.107	484	528	543	BV 2	21195	682512	0.16%	0.088%
4	8.314	892	914	1031	BV 2	534759	47938544	10.91%	6.164%
5	9.469	1077	1115	1168	BV 2	77441	6064485	1.38%	0.780%
6	10.315	1207	1263	1282	PV 4	22469	2019572	0.46%	0.260%
7	10.475	1282	1291	1317	VB 5	23860	1160727	0.26%	0.149%
8	11.219	1373	1421	1506	BV 3	47363	6443493	1.47%	0.828%
9	14.824	1786	2051	2164	VV 2	878508	439270302	100.00%	56.480%
10	15.587	2164	2185	2426	VB 2	769861	116890887	26.61%	15.029%
11	19.513	2801	2871	2998	BV 2	498532	67728842	15.42%	8.708%
12	20.286	2998	3006	3039	VB 2	14364	1196912	0.27%	0.154%
13	24.504	3611	3743	3796	BB 9	45842	6921397	1.58%	0.890%
14	25.424	3796	3904	3949	BV 9	36288	6682504	1.52%	0.859%
15	25.792	3949	3968	4000	VB 9	24018	1490906	0.34%	0.192%
16	26.326	4025	4061	4074	BV 5	120707	5232727	1.19%	0.673%
17	26.474	4074	4087	4115	VB 3	112151	5588634	1.27%	0.719%
18	27.162	4188	4207	4220	VV 3	24312	1548717	0.35%	0.199%
19	27.315	4220	4234	4252	VV 3	17741	1785211	0.41%	0.230%
20	27.554	4252	4276	4286	VV 9	59071	3839657	0.87%	0.494%
21	27.669	4286	4296	4346	VB 9	61898	5301420	1.21%	0.682%
22	28.102	4348	4372	4398	BV 9	31224	2056243	0.47%	0.264%
23	35.574	5581	5678	5738	BB 9	17016	4376064	1.00%	0.563%
24	39.438	6151	6353	6614	BB 7	44956	27846163	6.34%	3.580%
25	44.475	6932	7233	7339	VV 5	16037	8102433	1.84%	1.042%
26	63.083	10090	10485	10520	PV 5	1675	1886696	0.43%	0.243%
27	65.485	10520	10905	10940	VV 5	3752	4019382	0.92%	0.517%

Sum of corrected areas: 777749204

File
Operator :
Acquired : 7 Mar 2019 9:54 using AcqMethod FOMETHOD.M
Instrument : 5975 MSD
Sample Name: Apiary A 6
Misc Info :
Vial Number: 12

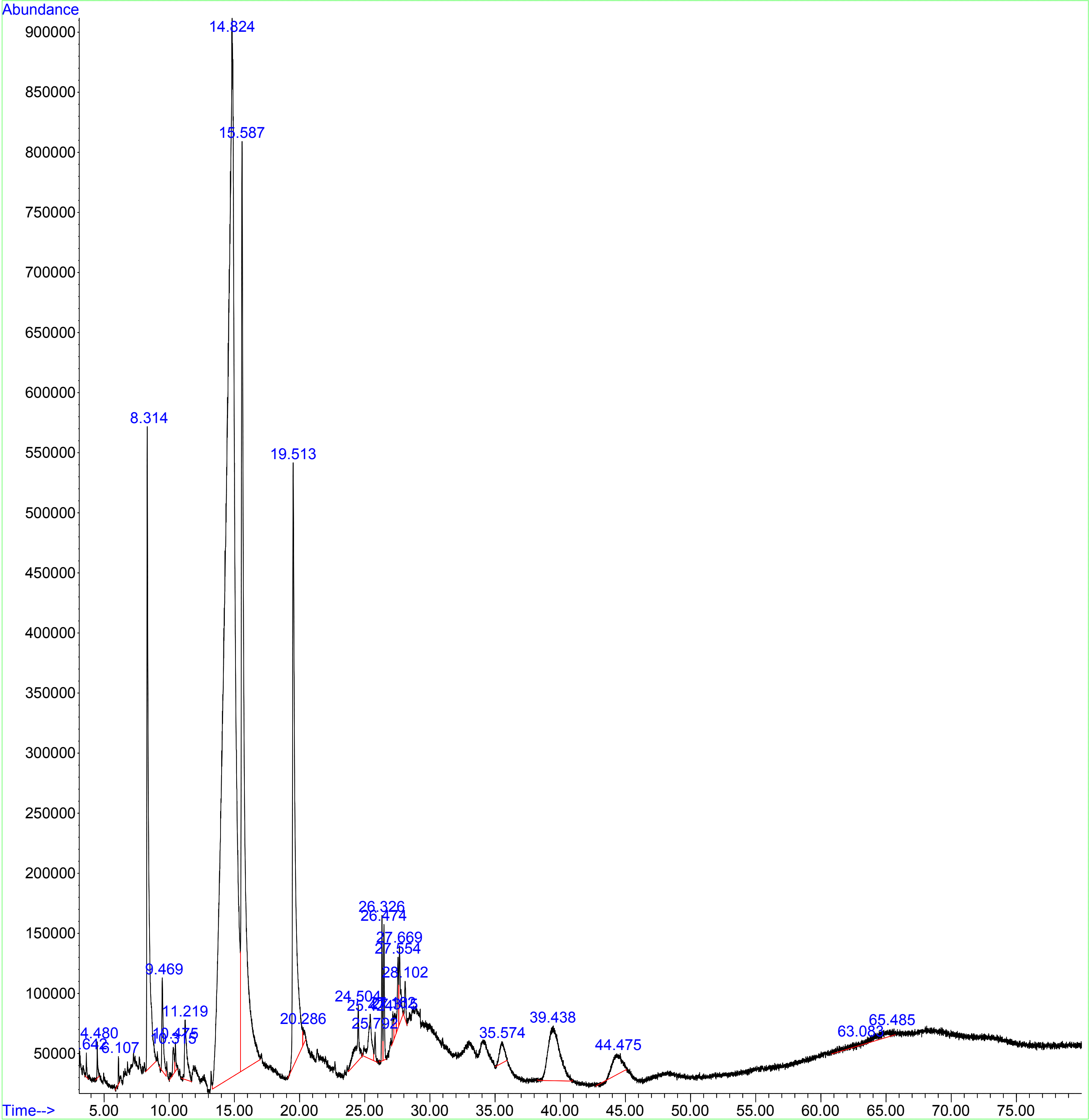

Acq On : 7 Mar 2019 11:24
Operator :
Sample : Apiary A 15
Misc :
ALS Vial : 13 Sample Multiplier: 1

Integration Parameters: autoint1.e
Integrator: ChemStation

peak #	R.T. min	first scan	max scan	last scan	PK TY	peak height	corr. area	corr. % max.	% of total
1	4.482	180	244	273	BB 5	18127	815760	0.33%	0.223%
2	6.113	505	529	539	BV 2	15267	301148	0.12%	0.082%
3	7.265	657	730	747	BV 4	11227	580681	0.24%	0.159%
4	8.323	900	915	1002	BV 2	242584	21797659	8.85%	5.971%
5	9.465	1074	1115	1168	BV 4	38231	2559091	1.04%	0.701%
6	11.216	1402	1421	1491	BB 4	24632	2869301	1.17%	0.786%
7	14.477	1783	1991	2160	BV 2	543918	246253203	100.00%	67.455%
8	15.583	2160	2184	2369	VB 3	414570	60205299	24.45%	16.492%
9	19.515	2752	2871	2993	BB 3	199431	27680478	11.24%	7.582%
10	24.504	3537	3743	3771	BB 3	9954	1849192	0.75%	0.507%
11	27.554	4132	4276	4286	BV 3	7349	153169	0.06%	0.042%

Sum of corrected areas: 365064981

File
Operator :
Acquired : 7 Mar 2019 11:24 using AcqMethod FOMETHOD.M
Instrument : 5975 MSD
Sample Name: Apiary A 15
Misc Info :
Vial Number: 13

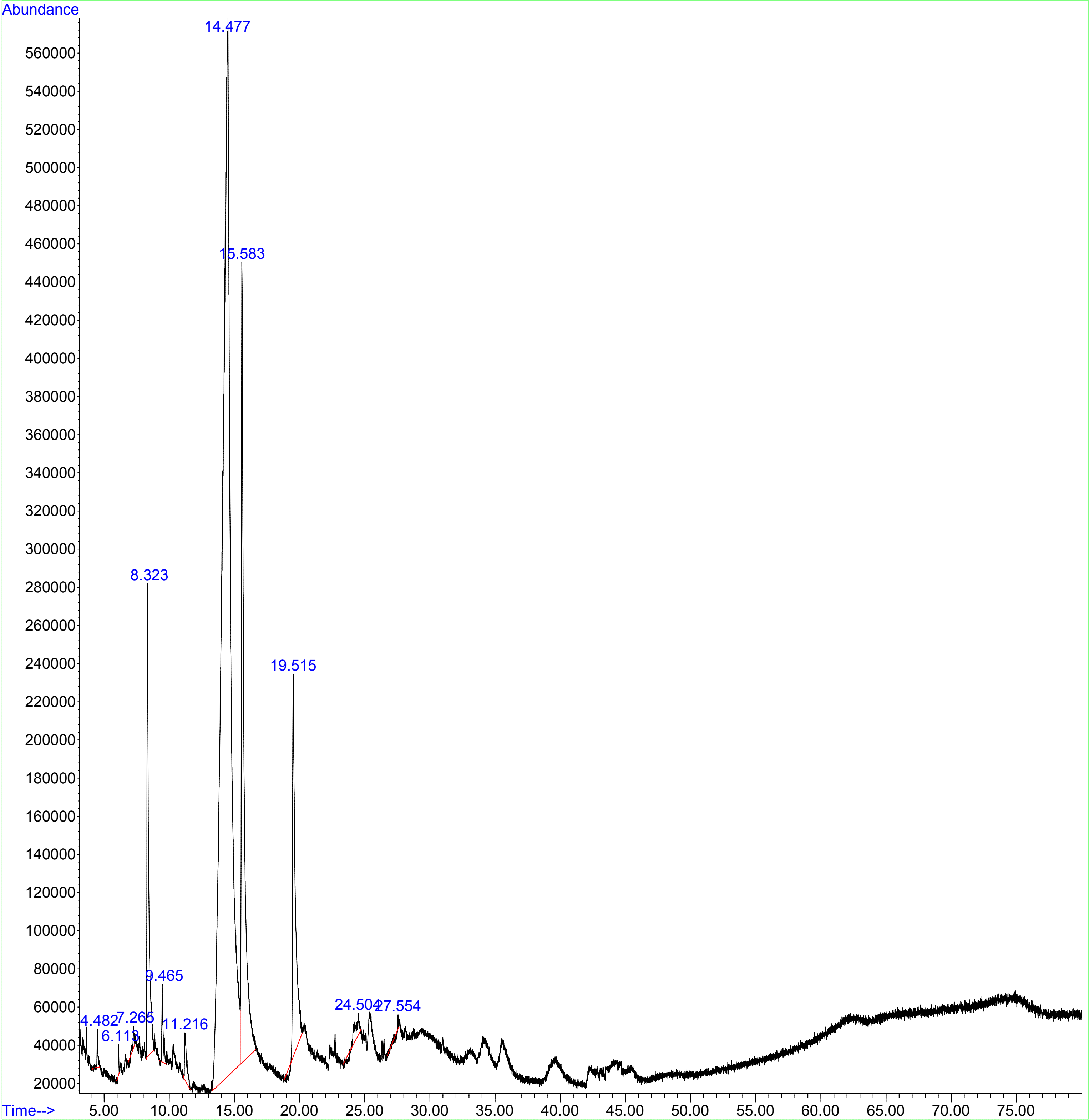

Acq On : 7 Mar 2019 12:53
Operator :
Sample : Apiary A 16
Misc :
ALS Vial : 14 Sample Multiplier: 1

Integration Parameters: autoint1.e
Integrator: ChemStation

peak #	R.T. min	first scan	max scan	last scan	PK TY	peak height	corr. area	corr. % max.	% of total
1	4.480	198	244	269	BV	38309	1333913	0.42%	0.240%
2	6.112	505	529	543	BV 3	21156	558367	0.17%	0.100%
3	6.624	577	618	638	BV 3	17550	920199	0.29%	0.165%
4	7.267	679	731	737	BV 8	20265	1422348	0.44%	0.256%
5	7.389	737	752	797	VB 6	28235	2735157	0.85%	0.491%
6	7.719	797	809	862	BB 2	24910	1650267	0.51%	0.297%
7	8.319	897	914	984	PV 2	465718	35818802	11.17%	6.436%
8	8.738	984	988	1037	VV 2	23536	2091743	0.65%	0.376%
9	9.476	1101	1117	1166	BV 3	50390	4161931	1.30%	0.748%
10	11.221	1390	1422	1476	BB 3	22973	2040544	0.64%	0.367%
11	13.342	1780	1792	1803	VV 3	22303	1159264	0.36%	0.208%
12	14.638	1803	2019	2144	VV 4	700659	320551545	100.00%	57.597%
13	15.382	2144	2149	2160	VV	93101	4637036	1.45%	0.833%
14	15.593	2160	2186	2262	VV 2	615659	85440965	26.65%	15.352%
15	16.054	2262	2266	2427	VV 5	81127	18226577	5.69%	3.275%
16	19.519	2803	2872	3059	BB 2	229077	38758593	12.09%	6.964%
17	25.462	3857	3910	3950	BB 2	16468	2552114	0.80%	0.459%
18	26.331	4034	4062	4076	BV 6	25241	1007468	0.31%	0.181%
19	26.482	4076	4089	4123	VB 6	21462	1235787	0.39%	0.222%
20	27.556	4140	4276	4286	BV 6	11767	981929	0.31%	0.176%
21	27.691	4286	4300	4314	VV 6	10347	601136	0.19%	0.108%
22	34.248	5341	5446	5517	BB 6	17266	5157927	1.61%	0.927%
23	35.592	5576	5681	5843	BB 6	24870	9697805	3.03%	1.743%
24	39.591	6204	6380	6551	BB 5	24734	13803629	4.31%	2.480%

Sum of corrected areas: 556545046

File
Operator :
Acquired : 7 Mar 2019 12:53 using AcqMethod FOMETHOD.M
Instrument : 5975 MSD
Sample Name: Apiary A 16
Misc Info :
Vial Number: 14

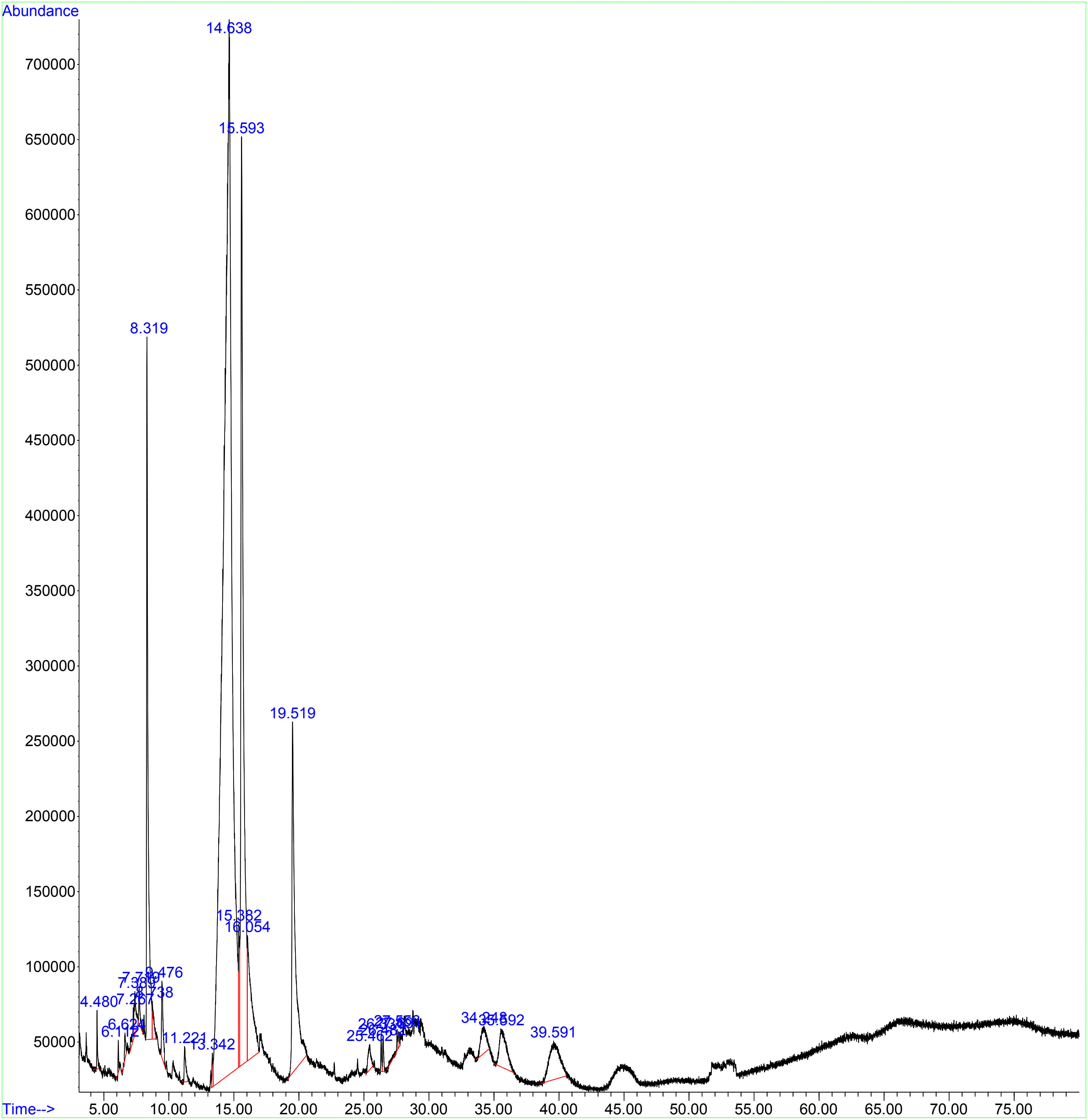

Acq On : 6 Mar 2019 18:59
Operator :
Sample : Apiary E 34 :
Misc
ALS Vial : 2 Sample Multiplier: 1

Integration Parameters: autoint1.e
Integrator: ChemStation

peak #	R.T. min	first scan	max scan	last scan	PK TY	peak height	corr. area	corr. % max.	% of total
1	3.628	82	95	117	BB	46097	970888	0.12%	0.061%
2	8.277	851	907	1024	BB 2	656461	50446426	6.11%	3.171%
3	9.434	1084	1109	1157	BB 2	96870	6004038	0.73%	0.377%
4	13.189	1737	1766	1773	BV 2	49725	2288441	0.28%	0.144%
5	15.075	1773	2095	2146	VV 4	1585728	825358655	100.00%	51.888%
6	15.483	2146	2166	2321	VB 2	2360726	169505282	20.54%	10.656%
7	19.210	2779	2818	2838	BV 7	132916	12048823	1.46%	0.757%
8	19.429	2838	2856	2967	VV	1137570	87210643	10.57%	5.483%
9	20.170	2967	2985	3044	VB 2	79135	7832810	0.95%	0.492%
10	22.683	3265	3425	3472	BB 2	38620	10178944	1.23%	0.640%
11	23.316	3472	3535	3701	BB 5	284813	67450636	8.17%	4.240%
12	25.125	3765	3852	3863	BV 8	118731	9713122	1.18%	0.611%
13	26.285	3863	4054	4069	VV 7	447403	205081498	24.85%	12.893%
14	26.412	4069	4076	4232	VB 8	417568	61377903	7.44%	3.859%
15	33.383	5163	5295	5369	BB 8	75769	16196442	1.96%	1.018%
16	34.631	5459	5513	5587	BB 7	284080	31133085	3.77%	1.957%
17	37.867	6018	6078	6160	BB 3	190267	27865494	3.38%	1.752%

Sum of corrected areas: 1590663129

Acq On : 6 Mar 2019 20:29
Operator :
Sample : Apiary E 36 :
Misc
ALS Vial : 3 Sample Multiplier: 1

Integration Parameters: autoint1.e
Integrator: ChemStation

peak #	R.T. min	first scan	max scan	last scan	PK TY	peak height	corr. area	corr. % max.	% of total
1	3.630	88	95	114	BV	31648	714761	0.10%	0.055%
2	8.279	880	907	960	BV 2	231018	14635115	2.04%	1.131%
3	9.438	1061	1110	1161	BB 3	76229	4775206	0.66%	0.369%
4	13.190	1748	1766	1768	BV 4	30247	829564	0.12%	0.064%
5	14.980	1768	2079	2143	VV 3	1467663	718892441	100.00%	55.560%
6	15.363	2143	2145	2149	VV	68253	1192421	0.17%	0.092%
7	15.488	2149	2167	2310	VV 2	1351425	102648202	14.28%	7.933%
8	19.226	2793	2821	2840	BV 2	86156	7545099	1.05%	0.583%
9	19.440	2840	2858	2972	VV 2	862213	75776579	10.54%	5.856%
10	20.199	2972	2991	3057	VB 2	72907	8370395	1.16%	0.647%
11	23.427	3495	3555	3635	BV 5	117960	23951379	3.33%	1.851%
12	25.148	3835	3856	3888	PV 4	136486	11456053	1.59%	0.885%
13	25.748	3888	3960	3963	VV 4	199979	28206053	3.92%	2.180%
14	25.969	3963	3999	4001	VV 8	246649	28224891	3.93%	2.181%
15	26.130	4001	4027	4032	VV 8	270642	27126303	3.77%	2.096%
16	26.292	4032	4055	4066	VV 8	334703	35040869	4.87%	2.708%
17	26.433	4066	4080	4254	VV 9	386536	96412686	13.41%	7.451%
18	27.507	4254	4268	4279	PV 7	48053	1874492	0.26%	0.145%
19	33.486	5240	5313	5398	BV 7	82052	16082823	2.24%	1.243%
20	34.763	5488	5536	5630	BV 7	302019	41579170	5.78%	3.213%
21	38.091	6043	6118	6246	BV 3	257571	48567218	6.76%	3.754%

Sum of corrected areas: 1293901721

File
Operator :
Acquired : 6 Mar 2019 20:29 using AcqMethod FOMETHOD.M
Instrument : 5975 MSD
Sample Name: Apiary E 36
Misc Info :
Vial Number: 3

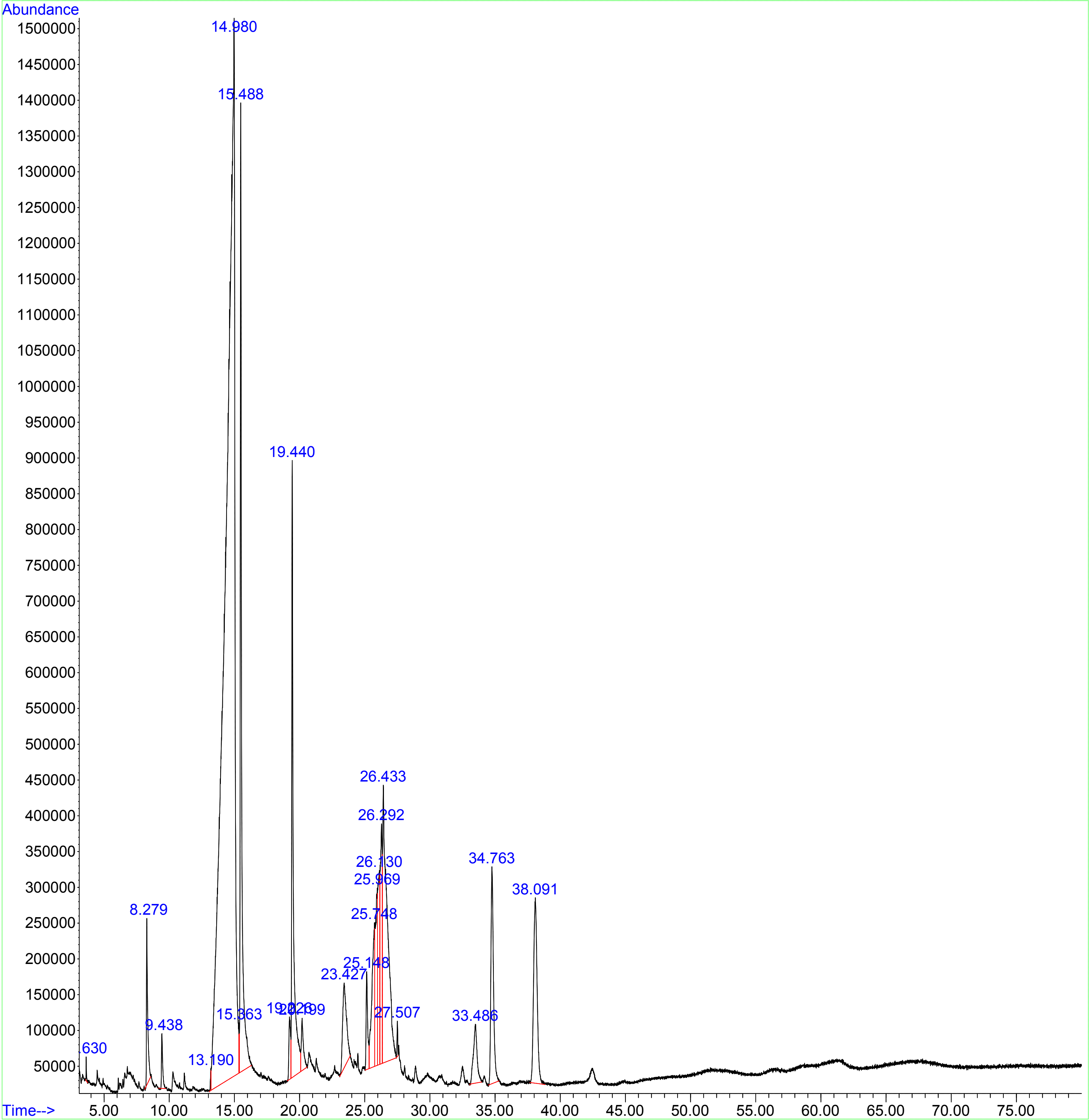

Acq On : 6 Mar 2019 21:58
Operator :
Sample : Apiary E 38 :
Misc
ALS Vial : 4 Sample Multiplier: 1

Integration Parameters: autoint1.e
Integrator: ChemStation

peak #	R.T. min	first scan	max scan	last scan	PK TY	peak height	corr. area	corr. % max.	% of total
1	6.789	637	647	673	VB 5	40386	1215824	0.08%	0.055%
2	8.283	889	908	1021	PV	476661	34558457	2.24%	1.551%
3	9.438	1073	1110	1163	BB 2	122251	7247841	0.47%	0.325%
4	10.287	1223	1258	1308	BV 5	43902	4135729	0.27%	0.186%
5	15.522	1774	2173	2416	VV 5	4522553	1544467611	100.00%	69.335%
6	17.051	2416	2440	2481	VB 9	17465	1964333	0.13%	0.088%
7	19.308	2777	2835	2842	BV 10	94220	7119025	0.46%	0.320%
8	19.464	2842	2862	2974	VV	1643202	146948013	9.51%	6.597%
9	20.224	2974	2995	3069	VV 3	111245	15123466	0.98%	0.679%
10	23.542	3499	3575	3727	BV 5	131628	41423009	2.68%	1.860%
11	24.483	3727	3739	3778	VB 4	79408	3886815	0.25%	0.174%
12	25.188	3840	3863	3891	BV 6	185906	14957903	0.97%	0.671%
13	25.402	3891	3900	3918	VV 9	53671	2708214	0.18%	0.122%
14	26.296	3918	4056	4068	VV 8	292725	58843898	3.81%	2.642%
15	26.440	4068	4081	4103	VV 5	357165	29539972	1.91%	1.326%
16	27.173	4103	4210	4257	VV 7	308217	139423124	9.03%	6.259%
17	27.516	4257	4269	4353	VV 7	422484	47313863	3.06%	2.124%
18	33.559	5255	5325	5416	BV 7	83178	17500935	1.13%	0.786%
19	34.876	5495	5556	5706	BB 7	416599	71659587	4.64%	3.217%
20	38.246	6049	6145	6268	BB 4	160330	37515705	2.43%	1.684%

Sum of corrected areas: 2227553325

File
Operator :
Acquired : 6 Mar 2019 21:58 using AcqMethod FOMETHOD.M
Instrument : 5975 MSD
Sample Name: Apiary E 38
Misc Info :
Vial Number: 4

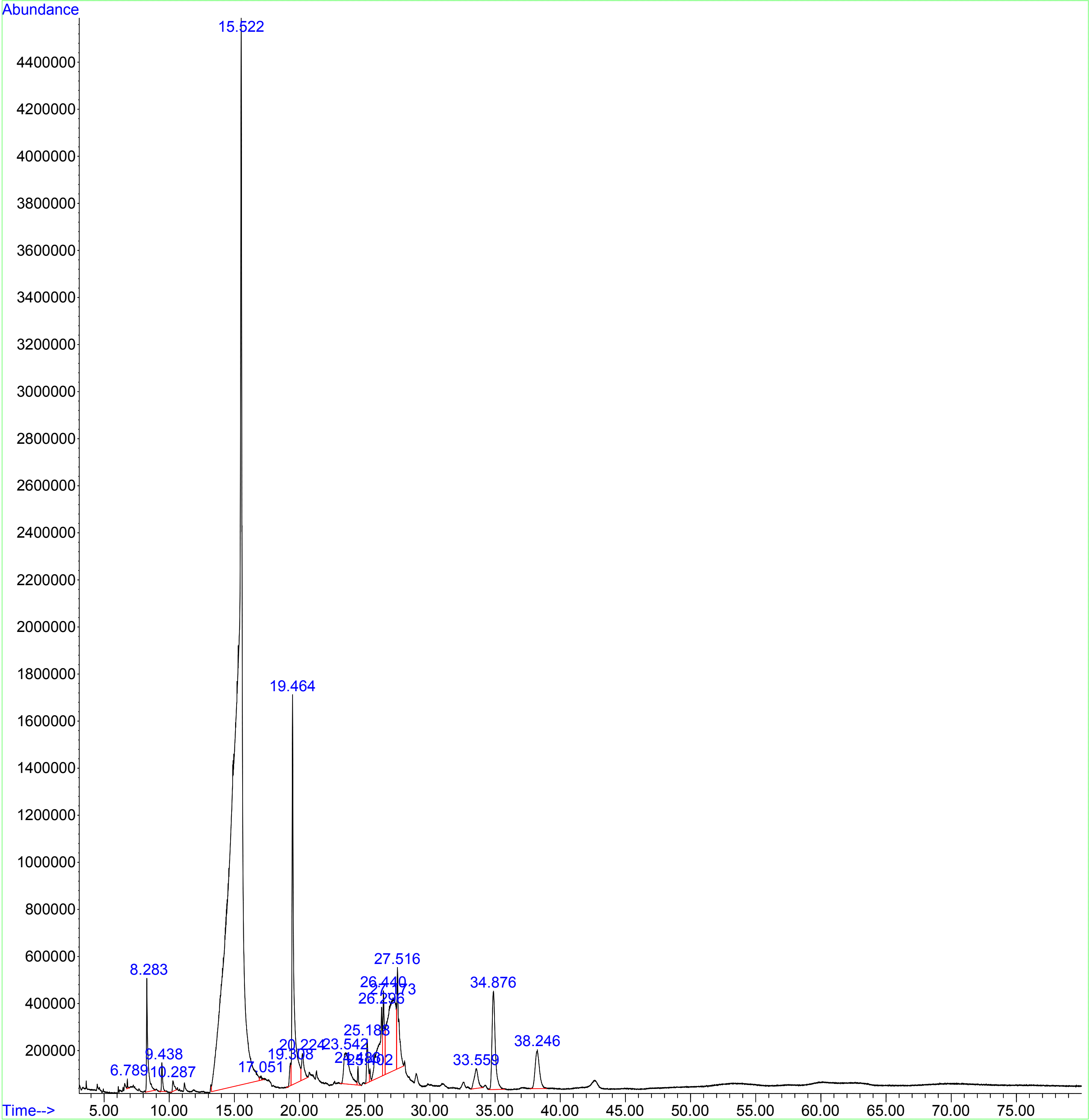

Acq On : 6 Mar 2019 23:28
Operator :
Sample : Apiary E 45 :
Misc
ALS Vial : 5 Sample Multiplier: 1

Integration Parameters: autoint1.e
Integrator: ChemStation

peak #	R.T. min	first scan	max scan	last scan	PK TY	peak height	corr. area	corr. % max.	% of total
1	8.290	889	909	1019	BB 3	350095	27078630	8.62%	3.782%
2	9.450	1074	1112	1166	BV 3	66558	4681217	1.49%	0.654%
3	14.456	1774	1987	2147	VV 3	784026	314281059	100.00%	43.897%
4	15.511	2147	2171	2342	VB 2	841848	76206420	24.25%	10.644%
5	19.456	2735	2861	2979	BV 2	485097	55949945	17.80%	7.815%
6	23.655	3445	3595	3697	PB 5	47631	14701001	4.68%	2.053%
7	26.299	3924	4057	4068	PV 8	108841	17231542	5.48%	2.407%
8	26.439	4068	4081	4105	VV 9	120097	11550787	3.68%	1.613%
9	27.110	4105	4198	4228	VV 9	104523	39364262	12.53%	5.498%
10	27.506	4228	4268	4279	VV 9	87616	14333134	4.56%	2.002%
11	27.630	4279	4289	4353	VV 9	84195	9822329	3.13%	1.372%
12	33.668	5265	5345	5428	BB 9	62960	14424323	4.59%	2.015%
13	34.985	5513	5575	5743	BB 9	393038	83353366	26.52%	11.642%
14	38.399	6071	6171	6313	BB 4	117379	32977036	10.49%	4.606%

Sum of corrected areas: 715955049

H

File
Operator :
Acquired : 6 Mar 2019 23:28 using AcqMethod FOMETHOD.M
Instrument : 5975 MSD
Sample Name: Apiary E 45
Misc Info :
Vial Number: 5

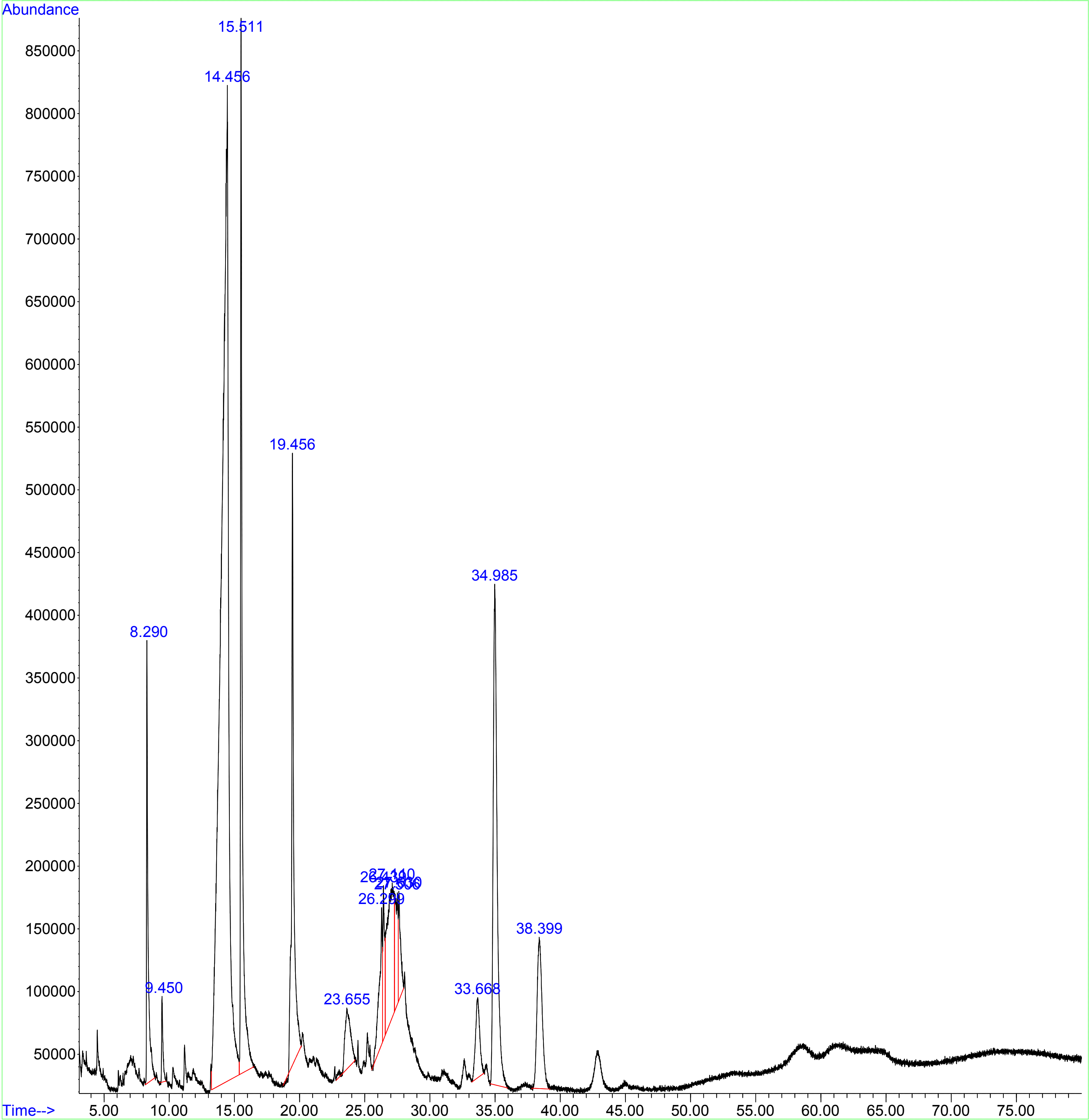

Acq On : 6 Mar 2019 17:30
Operator :
Sample : Apiary E 29 :
Misc
ALS Vial : 1 Sample Multiplier: 1

Integration Parameters: autoint1.e
Integrator: ChemStation

peak #	R.T. min	first scan	max scan	last scan	PK TY	peak height	corr. area	corr. % max.	% of total
1	3.646	80	98	115	BV 2	40337	1630100	0.24%	0.075%
2	4.194	115	193	238	VV 3	131453	30417717	4.45%	1.403%
3	4.483	238	244	259	VV 3	65711	3624922	0.53%	0.167%
4	4.644	259	272	333	VV 9	45045	5726966	0.84%	0.264%
5	5.169	333	364	381	PV 9	14504	786839	0.12%	0.036%
6	6.097	516	526	539	PV 8	23801	754020	0.11%	0.035%
7	6.651	597	623	637	VV 10	41397	3367617	0.49%	0.155%
8	6.791	637	647	687	VV 10	27422	2064350	0.30%	0.095%
9	7.375	736	749	759	VV 10	19612	1117311	0.16%	0.052%
10	7.693	759	805	817	VV 5	46545	5694424	0.83%	0.263%
11	7.920	817	845	848	VV 4	57483	5470839	0.80%	0.252%
12	7.955	848	851	856	VV 4	59927	1623437	0.24%	0.075%
13	8.093	856	875	878	VV 5	69067	4541289	0.66%	0.209%
14	8.121	878	880	894	VV	67404	2311855	0.34%	0.107%
15	8.289	894	909	959	VV 3	402433	31000652	4.54%	1.430%
16	8.587	959	961	993	VV 3	48464	3834414	0.56%	0.177%
17	9.445	1092	1111	1141	PV 4	67071	4357935	0.64%	0.201%
18	10.292	1234	1259	1276	PV 4	18739	1085649	0.16%	0.050%
19	11.165	1366	1412	1515	BV 7	51624	7407273	1.08%	0.342%
20	12.760	1659	1691	1703	PV 6	13377	811420	0.12%	0.037%
21	13.125	1703	1754	1796	VV 4	282326	41598956	6.09%	1.919%
22	14.227	1796	1947	1968	VV 5	1104491	238440534	34.90%	10.998%
23	15.227	1968	2122	2138	VV	1832773	683211048	100.00%	31.514%
24	15.465	2138	2163	2210	PV	1899139	104340410	15.27%	4.813%
25	15.749	2210	2213	2237	VV 4	58486	3374011	0.49%	0.156%
26	15.928	2237	2244	2258	VV 4	23680	1334279	0.20%	0.062%
27	16.014	2258	2259	2267	VV 8	12131	201254	0.03%	0.009%
28	16.417	2278	2330	2337	PV 6	18941	-619522	-0.09%	-0.029%
29	16.837	2337	2403	2405	PV 6	7629	-1052180	-0.15%	-0.049%
30	17.202	2405	2467	2492	PV 4	415554	48417120	7.09%	2.233%
31	17.605	2492	2537	2595	VV 4	429102	65156336	9.54%	3.005%
32	18.159	2595	2634	2643	VV 10	50402	4810050	0.70%	0.222%
33	18.505	2643	2695	2698	VV 9	34205	4221843	0.62%	0.195%
34	18.633	2698	2717	2735	VV 5	110144	8477661	1.24%	0.391%
35	18.764	2735	2740	2756	VB 5	31048	1277675	0.19%	0.059%
36	19.135	2774	2805	2837	BV 7	192386	12696406	1.86%	0.586%
37	19.421	2837	2855	2958	VV 2	955264	69459076	10.17%	3.204%
38	20.147	2958	2982	3040	VV 3	81087	7942521	1.16%	0.366%
39	20.612	3040	3063	3087	PV 7	75024	4223040	0.62%	0.195%
40	21.273	3164	3178	3192	BV 7	27169	1347155	0.20%	0.062%
41	21.730	3218	3258	3287	PV 2	83151	9905393	1.45%	0.457%

42	21.981	3287	3302	3308	VV	8	34587	2232513	0.33%	0.103%
43	22.055	3308	3315	3347	VV	8	38232	2588205	0.38%	0.119%
44	23.019	3452	3483	3507	PV	3	730858	53176885	7.78%	2.453%
45	23.169	3507	3510	3541	VV	2	192466	12640355	1.85%	0.583%
46	23.411	3541	3552	3577	VV	2	51623	3281481	0.48%	0.151%
47	24.926	3653	3817	3836	PV	2	885648	113647824	16.63%	5.242%
48	25.100	3836	3847	3861	VV	8	572054	43907514	6.43%	2.025%
49	25.190	3861	3863	3866	VV	4	474054	8599397	1.26%	0.397%
50	25.602	3866	3935	3957	VV	6	719787	154654910	22.64%	7.134%
51	26.461	4071	4085	4105	PV	7	27611	1096541	0.16%	0.051%
52	27.553	4236	4276	4288	PV	10	43658	2444468	0.36%	0.113%
53	27.660	4288	4295	4323	VV	10	22968	1434267	0.21%	0.066%
54	28.091	4323	4370	4395	VV	10	19342	2008050	0.29%	0.093%
55	28.338	4395	4413	4415	VV	10	23791	1015004	0.15%	0.047%
56	28.533	4415	4447	4484	VV	3	126730	13434021	1.97%	0.620%
57	28.808	4484	4495	4517	VV	3	36936	1986049	0.29%	0.092%
58	29.433	4573	4604	4613	BV	3	40757	2497579	0.37%	0.115%
59	29.590	4613	4632	4644	VV	3	78848	6127528	0.90%	0.283%
60	29.762	4644	4662	4717	VV	3	130372	16578367	2.43%	0.765%
61	30.264	4723	4750	4770	PV	3	17818	1395002	0.20%	0.064%
62	30.773	4795	4839	4863	BV	3	25315	1728831	0.25%	0.080%
63	31.431	4898	4954	5041	BV	7	384018	42840231	6.27%	1.976%
64	32.303	5088	5106	5114	VV	7	27573	1618355	0.24%	0.075%
65	32.402	5114	5123	5162	VB	7	24260	1564760	0.23%	0.072%
66	32.829	5164	5198	5223	BV	7	27805	2898150	0.42%	0.134%
67	33.099	5223	5245	5255	VV	9	44026	2353073	0.34%	0.109%
68	33.236	5255	5269	5277	VV	8	43427	2441366	0.36%	0.113%
69	33.364	5277	5291	5322	VV	7	90915	5943351	0.87%	0.274%
70	34.089	5322	5418	5452	PV	8	100724	19913916	2.91%	0.919%
71	34.300	5452	5455	5461	VV	8	24219	672917	0.10%	0.031%
72	34.346	5461	5463	5481	VV	8	22575	834865	0.12%	0.039%
73	34.600	5481	5507	5540	PV	5	299966	21007256	3.07%	0.969%
74	35.404	5595	5648	5689	BV		646800	68625388	10.04%	3.165%
75	37.015	5817	5929	5945	BV		55107	10146389	1.49%	0.468%
76	37.117	5945	5947	5962	VV		47278	2588882	0.38%	0.119%
77	37.280	5962	5976	5978	VV		48232	2378977	0.35%	0.110%
78	37.486	5978	6012	6021	VV		61063	8063385	1.18%	0.372%
79	37.762	6021	6060	6272	VV	3	439125	78670087	11.51%	3.629%
80	39.671	6296	6394	6398	BV	3	8593	861214	0.13%	0.040%
81	39.714	6398	6401	6418	VB	3	7523	239587	0.04%	0.011%
82	40.434	6452	6527	6599	PV	2	79139	15236890	2.23%	0.703%
83	42.028	6765	6805	6809	BV	2	39574	2787804	0.41%	0.129%
84	42.064	6809	6812	6880	VB	3	40815	3393139	0.50%	0.157%

Sum of corrected areas: 2167946866

File
Operator :
Acquired : 7 Mar 2019 21:50 using AcqMethod FOMETHOD.M
Instrument : 5975 MSD
Sample Name: Apiary D 4
Misc Info :
Vial Number: 20

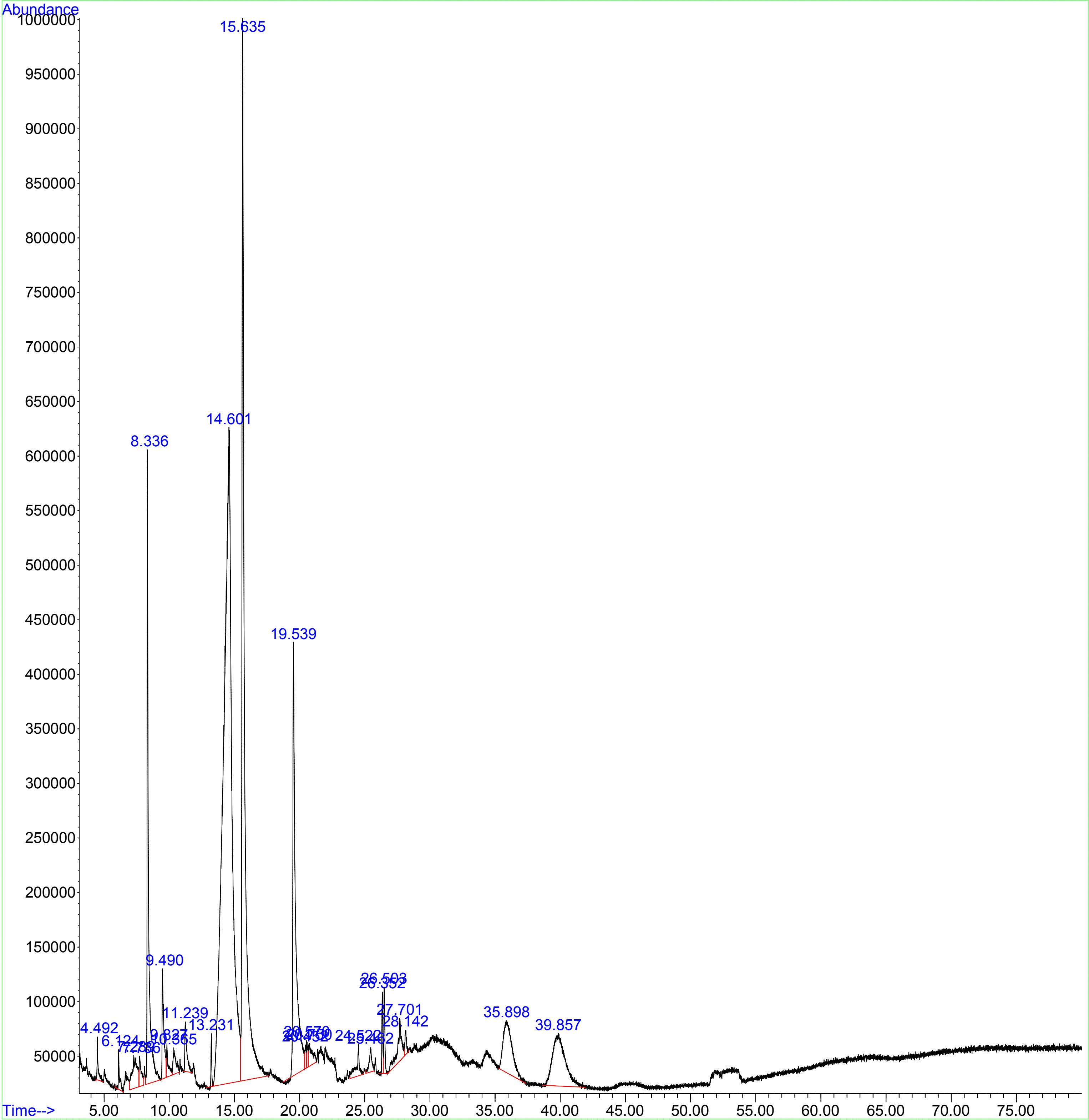

Acq On : 7 Mar 2019 21:50
Operator :
Sample : Apiary D 4
Misc :
ALS Vial : 20 Sample Multiplier: 1

Integration Parameters: autoint1.e
Integrator: ChemStation

peak #	R.T. min	first scan	max scan	last scan	PK TY	peak height	corr. area	corr. % max.	% of total
1	4.492	226	246	325	PV 4	35579	2274432	0.85%	0.364%
2	6.124	482	531	590	BV 4	30919	2285282	0.85%	0.366%
3	7.289	674	734	802	VV 4	27934	7718274	2.88%	1.235%
4	7.736	802	813	865	VV 3	27412	3174285	1.18%	0.508%
5	8.336	891	917	1092	VV 2	535064	42529464	15.85%	6.804%
6	9.490	1092	1119	1167	PV 2	95179	9106172	3.39%	1.457%
7	9.827	1167	1178	1248	VV 2	28517	2875543	1.07%	0.460%
8	10.365	1248	1272	1342	VV 9	21597	3506085	1.31%	0.561%
9	11.239	1392	1425	1515	BV 2	44226	4736640	1.76%	0.758%
10	13.231	1705	1773	1795	BV 3	45566	1620928	0.60%	0.259%
11	14.601	1795	2012	2164	VV 2	581951	268374605	100.00%	42.934%
12	15.635	2164	2193	2545	VV 2	968584	133269783	49.66%	21.320%
13	19.539	2731	2875	3023	BV 2	391795	56068749	20.89%	8.970%
14	20.452	3023	3035	3048	VV 2	19861	1504594	0.56%	0.241%
15	20.570	3048	3055	3071	VV 2	21107	1490099	0.56%	0.238%
16	20.750	3071	3087	3181	VV 2	20218	4138478	1.54%	0.662%
17	24.522	3630	3746	3814	VV 2	27383	4333258	1.61%	0.693%
18	25.462	3843	3910	3957	VV 2	21981	3078058	1.15%	0.492%
19	26.352	4014	4066	4079	BV 6	73509	3509225	1.31%	0.561%
20	26.503	4079	4092	4141	VV 5	79074	4514093	1.68%	0.722%
21	27.701	4141	4302	4361	PV 5	37185	8159124	3.04%	1.305%
22	28.142	4361	4379	4419	VV 5	21394	1859451	0.69%	0.297%
23	35.898	5630	5734	5984	BV 5	46087	22717897	8.46%	3.634%
24	39.857	6213	6426	6783	BB 5	46520	32240515	12.01%	5.158%

Sum of corrected areas: 625085035

Acq On : 7 Mar 2019 23:20
Operator :
Sample : Apiary D 7
Misc :
ALS Vial : 21 Sample Multiplier: 1

Integration Parameters: autoint1.e
Integrator: ChemStation

peak #	R.T. min	first scan	max scan	last scan	PK TY	peak height	corr. area	corr. % max.	% of total
1	3.654	86	99	122	PV	26313	640649	0.12%	0.057%
2	4.495	227	246	329	VV 7	22433	2102928	0.38%	0.187%
3	6.122	505	531	545	BV 3	20422	1035510	0.19%	0.092%
4	8.343	889	919	1069	VV 2	376265	32855785	5.93%	2.923%
5	9.489	1069	1119	1172	VV 2	119917	11516156	2.08%	1.025%
6	10.333	1242	1266	1342	VV 2	40754	6308344	1.14%	0.561%
7	11.243	1388	1425	1515	BV 2	27047	2644071	0.48%	0.235%
8	13.237	1708	1774	1798	BV 3	24250	856861	0.15%	0.076%
9	15.147	1798	2108	2172	PV 3	1049269	554299673	100.00%	49.315%
10	15.652	2172	2196	2424	VV	1568065	220360424	39.75%	19.605%
11	17.092	2424	2448	2555	VV 7	24842	5802455	1.05%	0.516%
12	19.552	2801	2878	3008	BV	737239	95419853	17.21%	8.489%
13	20.368	3008	3020	3181	VV	40038	11929011	2.15%	1.061%
14	21.395	3181	3200	3284	VB	19134	2153666	0.39%	0.192%
15	24.526	3511	3747	3868	BV 9	46905	19008070	3.43%	1.691%
16	25.456	3868	3909	4048	VV 9	51616	13493354	2.43%	1.200%
17	26.512	4048	4094	4144	VV 8	69807	6594339	1.19%	0.587%
18	27.612	4144	4286	4354	PV 8	35115	8205699	1.48%	0.730%
19	28.151	4354	4380	4461	VB 8	19312	2565124	0.46%	0.228%
20	31.072	4464	4891	5227	BV 10	29458	40266153	7.26%	3.582%
21	39.925	6190	6438	6577	BV 3	102550	65498333	11.82%	5.827%
22	40.799	6577	6591	6887	VB 5	45507	20452837	3.69%	1.820%

Sum of corrected areas: 1124009294

File
Operator :
Acquired : 7 Mar 2019 23:20 using AcqMethod FOMETHOD.M
Instrument : 5975 MSD
Sample Name: Apiary D 7
Misc Info :
Vial Number: 21

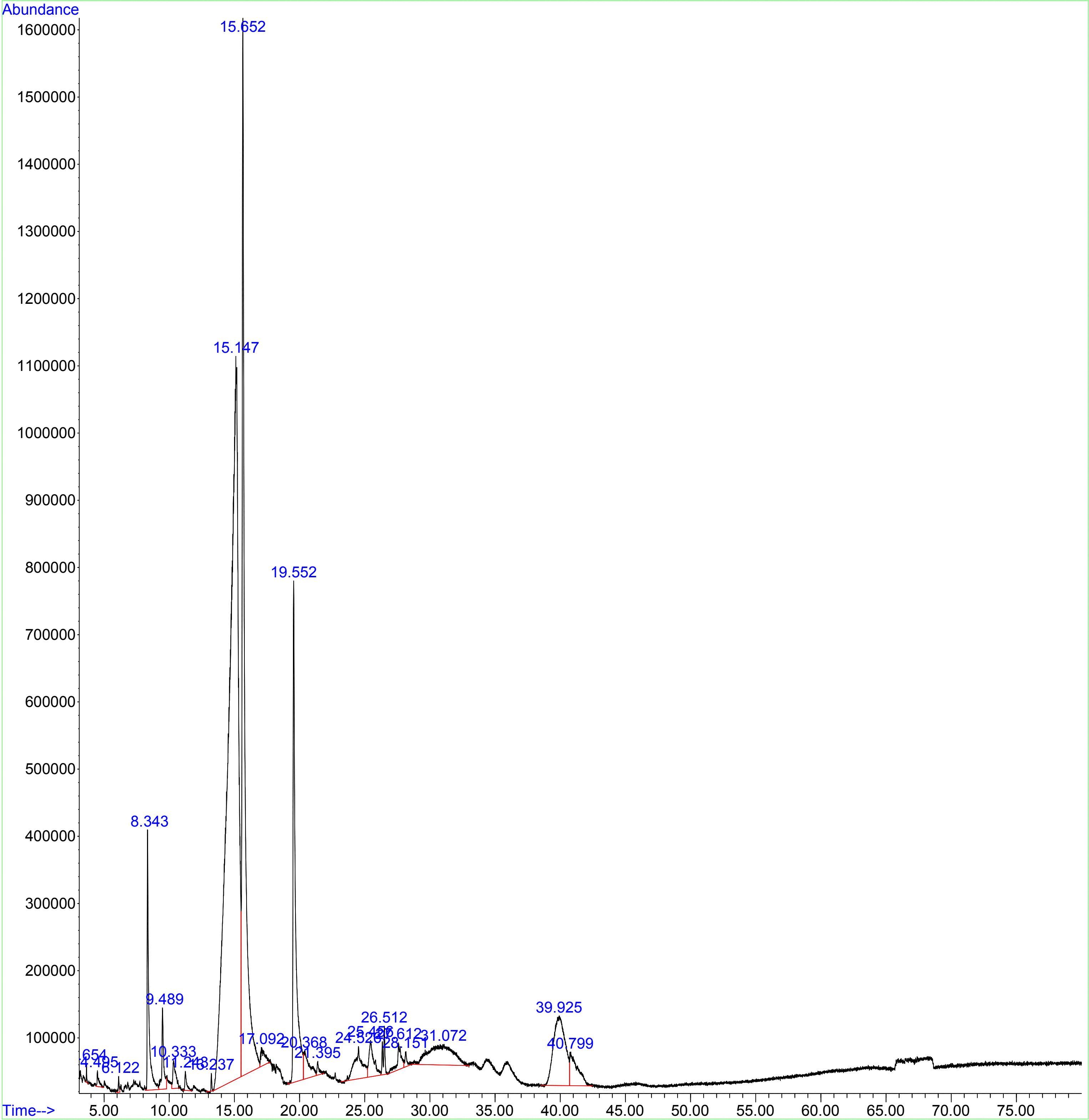

Acq On : 8 Mar 2019 00:49
Operator :
Sample : Apiary D 14 :
Misc : 22
ALS Vial Sample Multiplier: 1

Integration Parameters: autoint1.e
Integrator: ChemStation

peak #	R.T. min	first scan	max scan	last scan	PK TY	peak height	corr. area	corr. % max.	% of total
1	8.342	892	918	1038	PV 2	287847	23440200	2.48%	1.388%
2	9.485	1038	1118	1173	VV 2	100813	6391577	0.68%	0.378%
3	10.332	1221	1266	1316	BV 2	35764	3080175	0.33%	0.182%
4	14.588	1802	2010	2022	VV 2	687964	237940104	25.20%	14.086%
5	14.788	2022	2045	2051	VV 3	761944	69165396	7.32%	4.094%
6	15.661	2051	2198	2532	VV 6	2681902	944317142	100.00%	55.902%
7	19.567	2817	2880	3003	BV 2	1099488	141448956	14.98%	8.374%
8	20.382	3003	3023	3105	VB 6	55062	11620285	1.23%	0.688%
9	23.681	3552	3599	3617	BV 10	32386	2282867	0.24%	0.135%
10	24.272	3617	3702	3710	VV 5	53009	7285977	0.77%	0.431%
11	24.529	3710	3747	3771	VV 4	170532	15963493	1.69%	0.945%
12	24.695	3771	3776	3813	VV 4	51058	6406933	0.68%	0.379%
13	25.465	3813	3911	3960	VV 8	194893	39382735	4.17%	2.331%
14	25.828	3960	3974	4052	VV 8	106769	12556936	1.33%	0.743%
15	26.365	4052	4068	4082	VV 2	247602	12240429	1.30%	0.725%
16	26.516	4082	4095	4147	VB 2	261880	15656575	1.66%	0.927%
17	27.603	4210	4285	4296	BV 5	103911	4437375	0.47%	0.263%
18	27.700	4296	4302	4318	VV 7	59539	2696216	0.29%	0.160%
19	29.876	4547	4682	4688	PV 7	31301	11613172	1.23%	0.687%
20	31.440	4688	4955	5130	VB 8	46607	51214922	5.42%	3.032%
21	39.987	6270	6449	6860	BB 6	94040	70102773	7.42%	4.150%

Sum of corrected areas: 1689244239

File
Operator :
Acquired : 8 Mar 2019 00:49 using AcqMethod FOMETHOD.M
Instrument : 5975 MSD
Sample Name: Apiary D 14
Misc Info :
Vial Number: 22

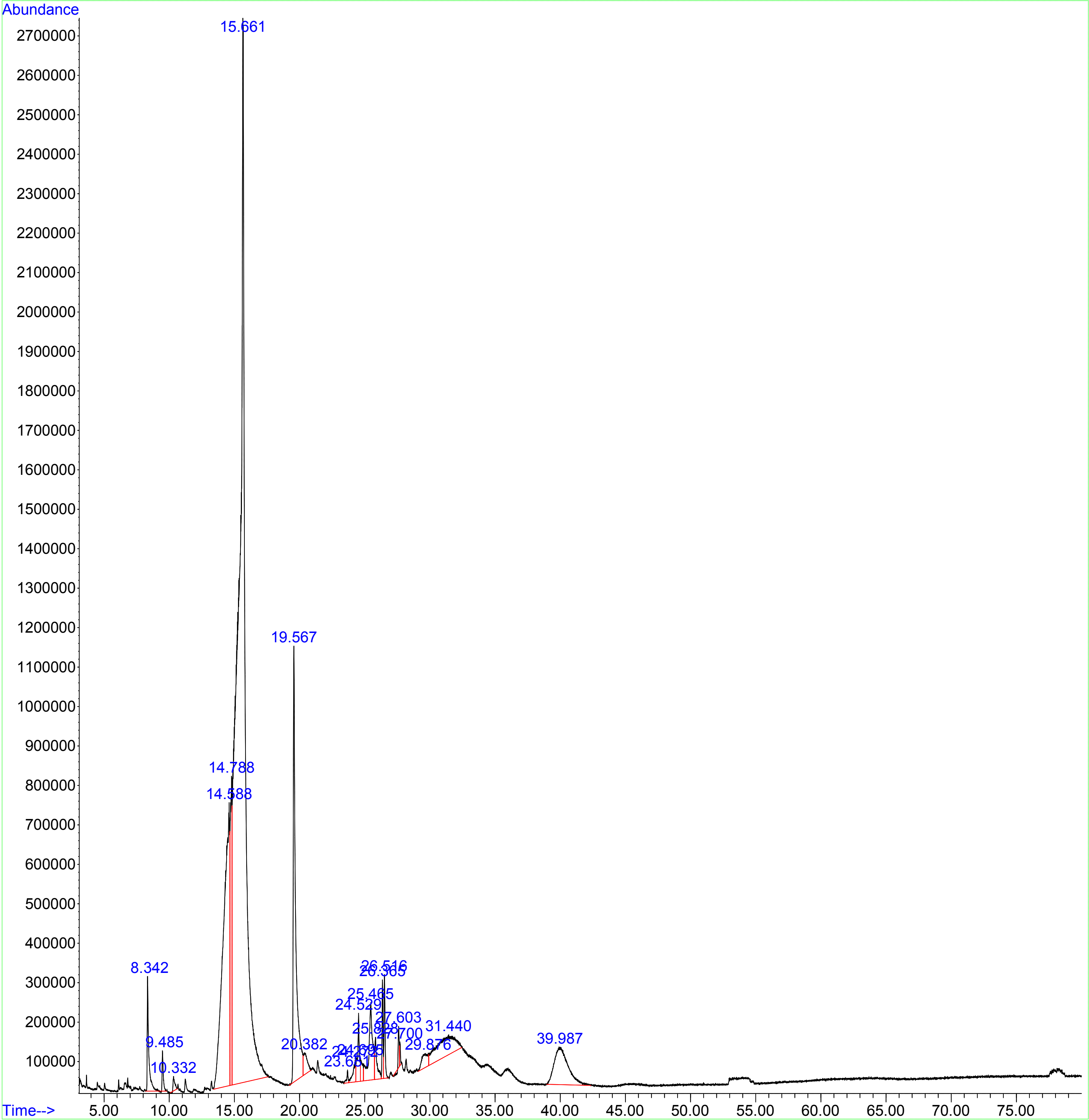

Acq On : 8 Mar 2019 2:19
Operator :
Sample : Apiary D 17 :
Misc : 23
ALS Vial Sample Multiplier: 1

Integration Parameters: autoint1.e
Integrator: ChemStation

peak #	R.T. min	first scan	max scan	last scan	PK TY	peak height	corr. area	corr. % max.	% of total
1	4.488	206	245	281	BB 3	25965	1298783	0.37%	0.176%
2	7.285	670	734	749	PV 3	10688	781646	0.22%	0.106%
3	8.347	891	919	1041	PV	629743	49180967	13.92%	6.668%
4	9.496	1076	1120	1173	BV 4	80273	5646072	1.60%	0.766%
5	11.247	1389	1426	1519	BB 3	31463	3291983	0.93%	0.446%
6	13.242	1739	1775	1799	BV 3	33575	1397323	0.40%	0.189%
7	14.596	1799	2011	2172	PV 3	784653	353385333	100.00%	47.914%
8	15.657	2172	2197	2439	VV 2	1465486	190148523	53.81%	25.782%
9	19.553	2780	2878	3010	BV 2	604916	86093423	24.36%	11.673%
10	20.414	3010	3028	3119	VV 2	32449	6385670	1.81%	0.866%
11	24.533	3733	3748	3797	VB 2	21405	1925310	0.54%	0.261%
12	26.362	4035	4068	4082	BV 7	37332	1855637	0.53%	0.252%
13	26.512	4082	4094	4132	VB 6	39341	2375293	0.67%	0.322%
14	36.038	5634	5759	5924	BB 6	34725	16028544	4.54%	2.173%
15	39.924	6288	6438	6624	BB 5	29548	17739726	5.02%	2.405%

Sum of corrected areas: 737534233

File
Operator :
Acquired : 8 Mar 2019 2:19 using AcqMethod FOMETHOD.M
Instrument : 5975 MSD
Sample Name: Apiary D 17
Misc Info :
Vial Number: 23

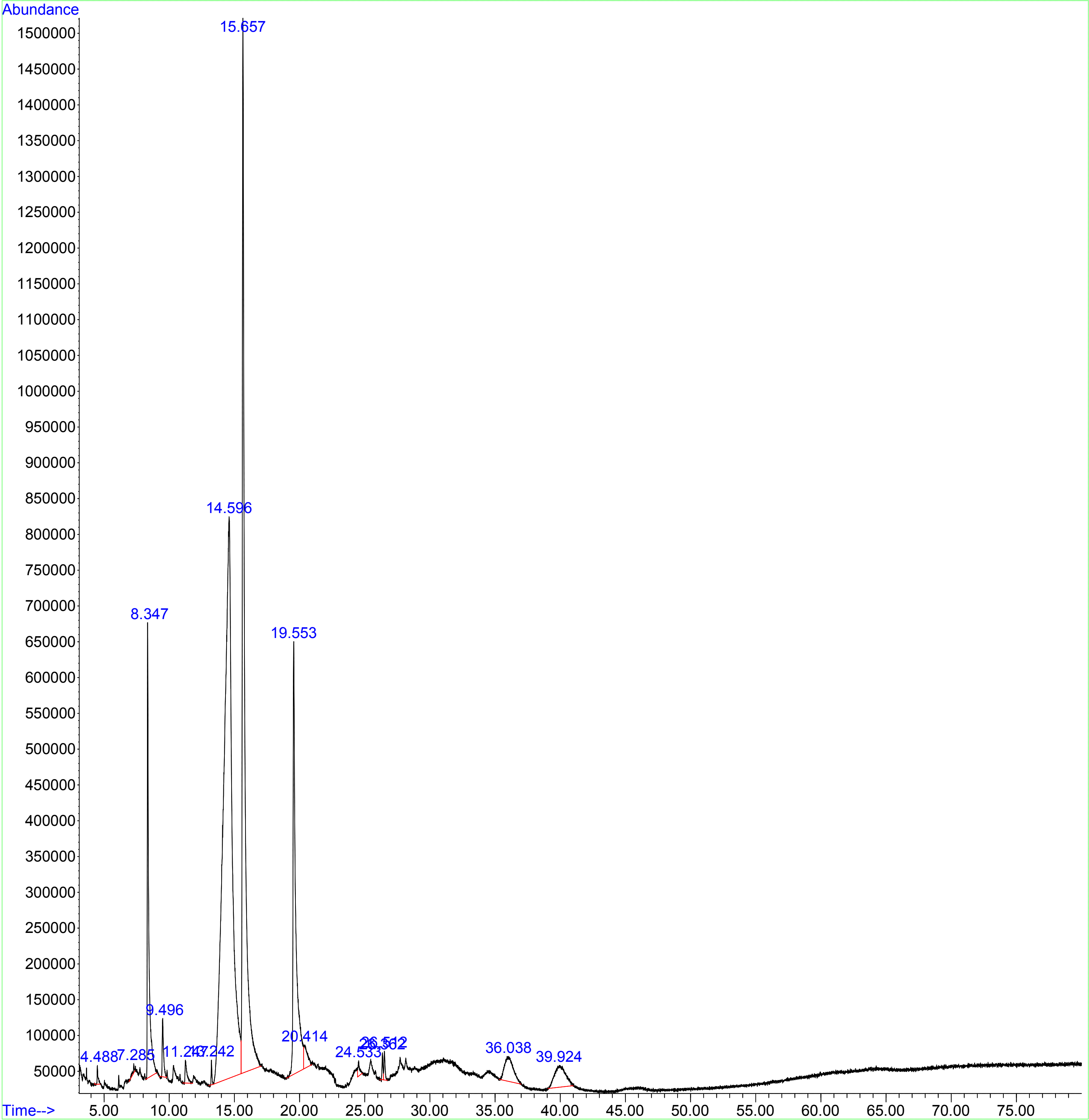

Acq On : 8 Mar 2019 3:48
Operator :
Sample : Apiary D
Misc 23 :
ALS Vial : 24 Sample Multiplier: 1

Integration Parameters: autoint1.e
Integrator: ChemStation

peak #	R.T. min	first scan	max scan	last scan	PK TY	peak height	corr. area	corr. % max.	% of total
1	4.483	226	244	254	BV 3	33808	969894	0.29%	0.127%
2	6.126	473	531	545	BV 3	25748	285023	0.09%	0.037%
3	7.291	716	735	749	VV 4	42667	3070401	0.93%	0.403%
4	7.411	749	756	764	VV 5	39232	1629710	0.50%	0.214%
5	7.521	764	775	795	VV 7	44811	3808104	1.16%	0.500%
6	7.762	795	817	854	VV	49412	5848446	1.78%	0.767%
7	8.348	887	919	1041	VV	864426	67164794	20.43%	8.812%
8	9.496	1093	1120	1169	BV	74764	5771518	1.76%	0.757%
9	11.252	1393	1427	1519	BV 3	40325	4213300	1.28%	0.553%
10	13.243	1737	1775	1794	BV 2	44568	1812061	0.55%	0.238%
11	14.676	1794	2025	2172	VV 3	886881	328781737	100.00%	43.136%
12	15.663	2172	2198	2446	VV	1492442	197346619	60.02%	25.892%
13	19.558	2775	2879	3119	BV 2	467706	77257792	23.50%	10.136%
14	24.536	3613	3749	3803	PB 2	22245	4854089	1.48%	0.637%
15	26.368	4052	4069	4082	PV 10	38404	1969842	0.60%	0.258%
16	26.520	4082	4095	4135	VV 7	40121	2747649	0.84%	0.360%
17	27.721	4135	4305	4353	PV 7	30589	7348809	2.24%	0.964%
18	28.160	4353	4382	4427	VV 7	21368	2721929	0.83%	0.357%
19	36.105	5623	5770	6003	BV 7	56859	28219558	8.58%	3.702%
20	40.010	6231	6453	6582	BB 5	31500	16377372	4.98%	2.149%

Sum of corrected areas: 762198646

File
Operator :
Acquired : 8 Mar 2019 3:48 using AcqMethod FOMETHOD.M
Instrument : 5975 MSD
Sample Name: Apiary D 23
Misc Info :
Vial Number: 24

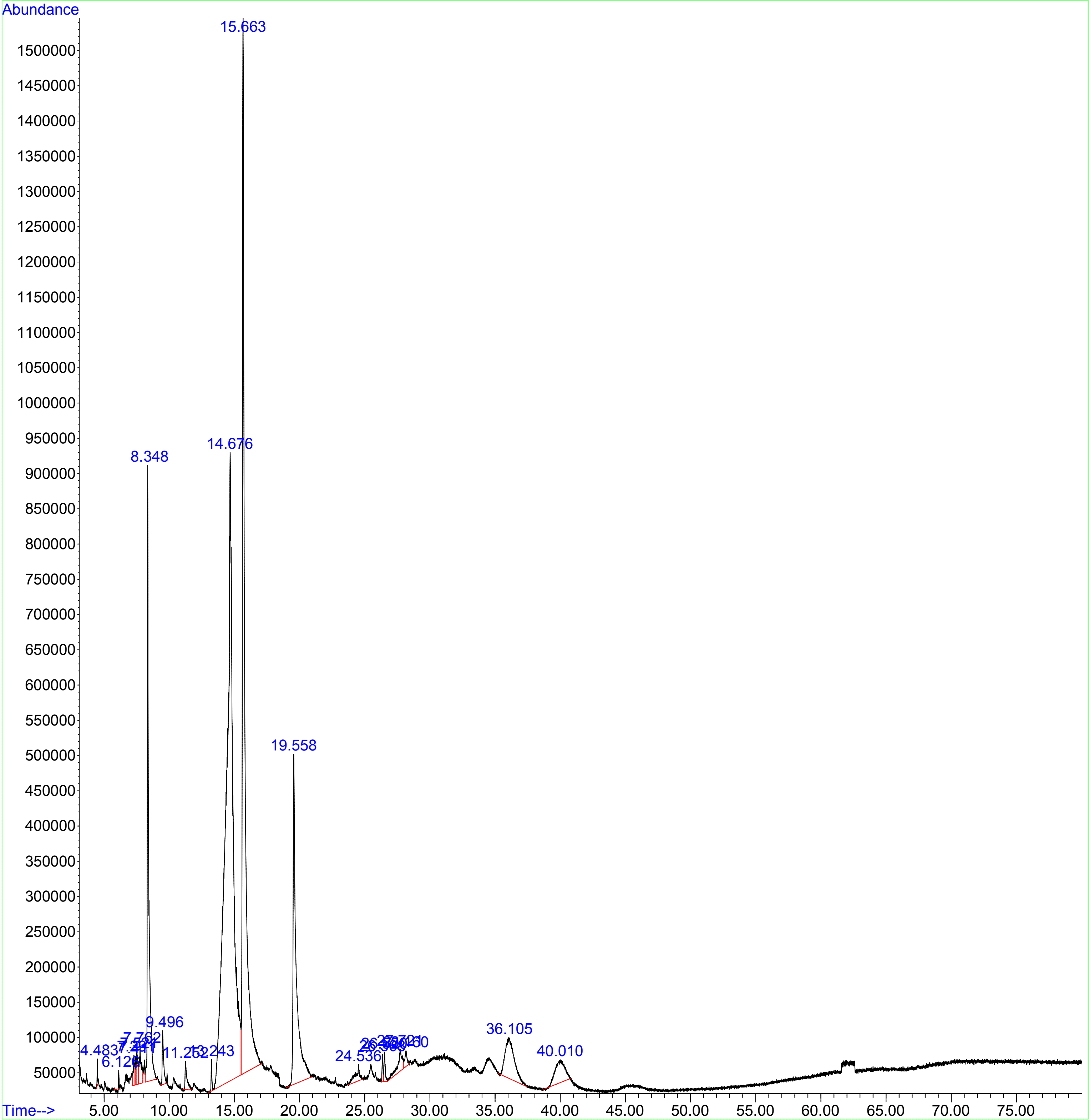

Acq On : 8 Mar 2019 5:17
Operator :
Sample : Apiary D 24 :
Misc : 25
ALS Vial Sample Multiplier: 1

Integration Parameters: autoint1.e
Integrator: ChemStation

peak #	R.T. min	first scan	max scan	last scan	PK TY	peak height	corr. area	corr. % max.	% of total
1	4.483	211	244	284	BB	80280	3124320	0.89%	0.388%
2	6.125	487	531	547	BV 2	46776	1215882	0.35%	0.151%
3	7.294	590	735	749	BV 7	18341	2016659	0.57%	0.250%
4	8.355	905	921	1092	VB 2	1575705	117090096	33.26%	14.538%
5	9.497	1093	1120	1170	BV 2	122531	8971594	2.55%	1.114%
6	9.836	1170	1180	1206	VB 3	50177	1982532	0.56%	0.246%
7	10.336	1236	1267	1306	BB 7	23105	2059984	0.59%	0.256%
8	11.253	1395	1427	1521	BB 2	102865	11026605	3.13%	1.369%
9	13.245	1752	1775	1790	BV 2	55714	2210248	0.63%	0.274%
10	13.393	1790	1801	1832	VV 2	48131	3104879	0.88%	0.386%
11	14.883	1832	2062	2175	VV 4	724582	352010016	100.00%	43.706%
12	15.677	2175	2200	2442	VV 2	1214979	168010477	47.73%	20.860%
13	19.567	2804	2880	3102	BB 3	666932	104753656	29.76%	13.006%
14	24.534	3544	3748	3797	BB 3	26420	6119923	1.74%	0.760%
15	26.371	4038	4069	4082	BV 7	44587	2171171	0.62%	0.270%
16	26.521	4082	4095	4124	VB 6	47223	2947313	0.84%	0.366%
17	27.729	4132	4307	4350	BB 6	21555	1642050	0.47%	0.204%
18	28.174	4351	4384	4420	BV 6	41018	3463652	0.98%	0.430%
19	28.494	4420	4440	4459	VV 6	17693	1246795	0.35%	0.155%
20	40.132	6287	6474	6571	BB 8	22490	10238166	2.91%	1.271%

Sum of corrected areas: 805406018

File
Operator :
Acquired : 8 Mar 2019 5:17 using AcqMethod FOMETHOD.M
Instrument : 5975 MSD
Sample Name: Apiary D 24
Misc Info :
Vial Number: 25

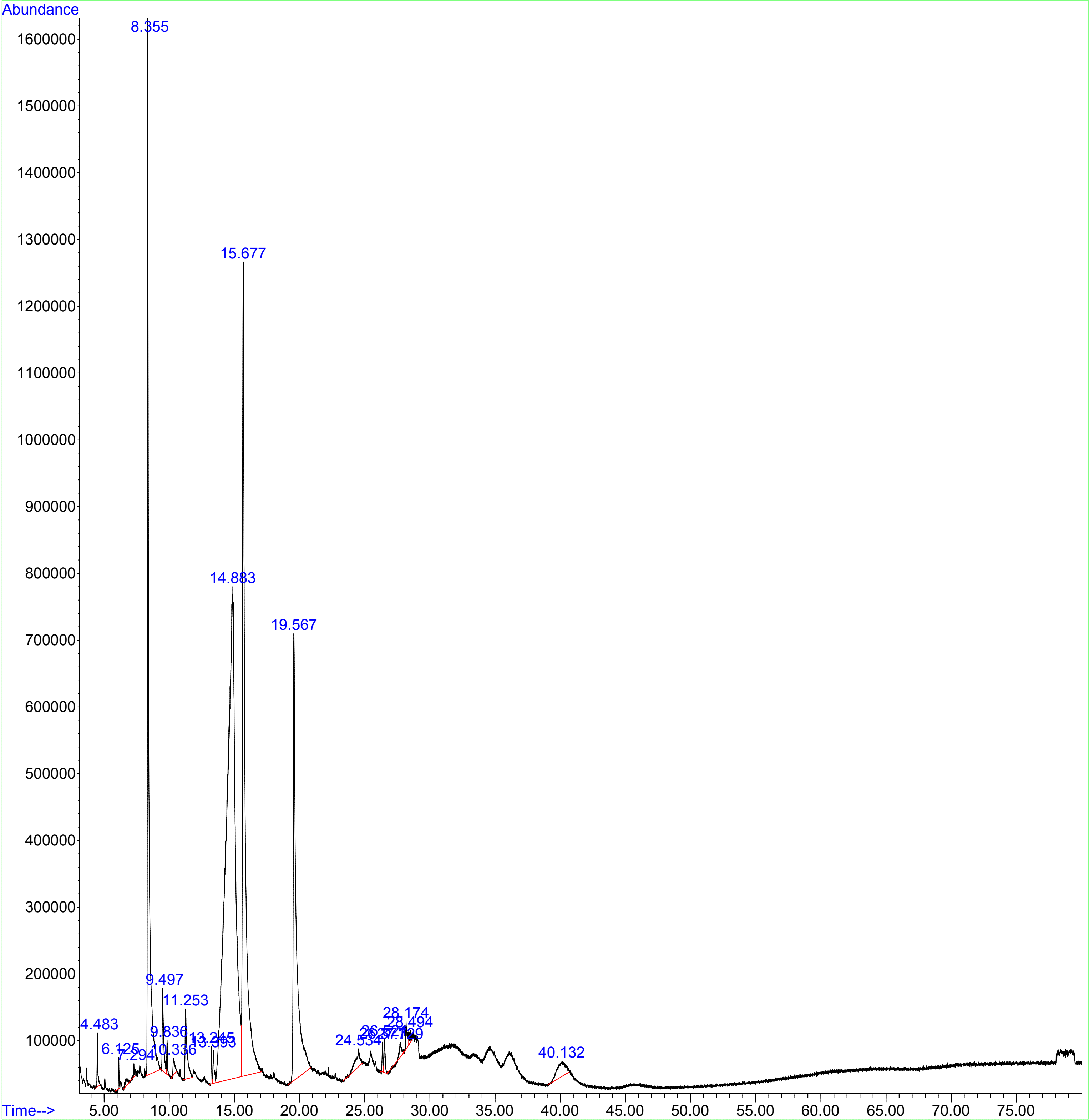

File
Operator :
Acquired : 7 Mar 2019 14:23 using AcqMethod FOMETHOD.M
Instrument : 5975 MSD
Sample Name: Apiary C 1
Misc Info :
Vial Number: 15

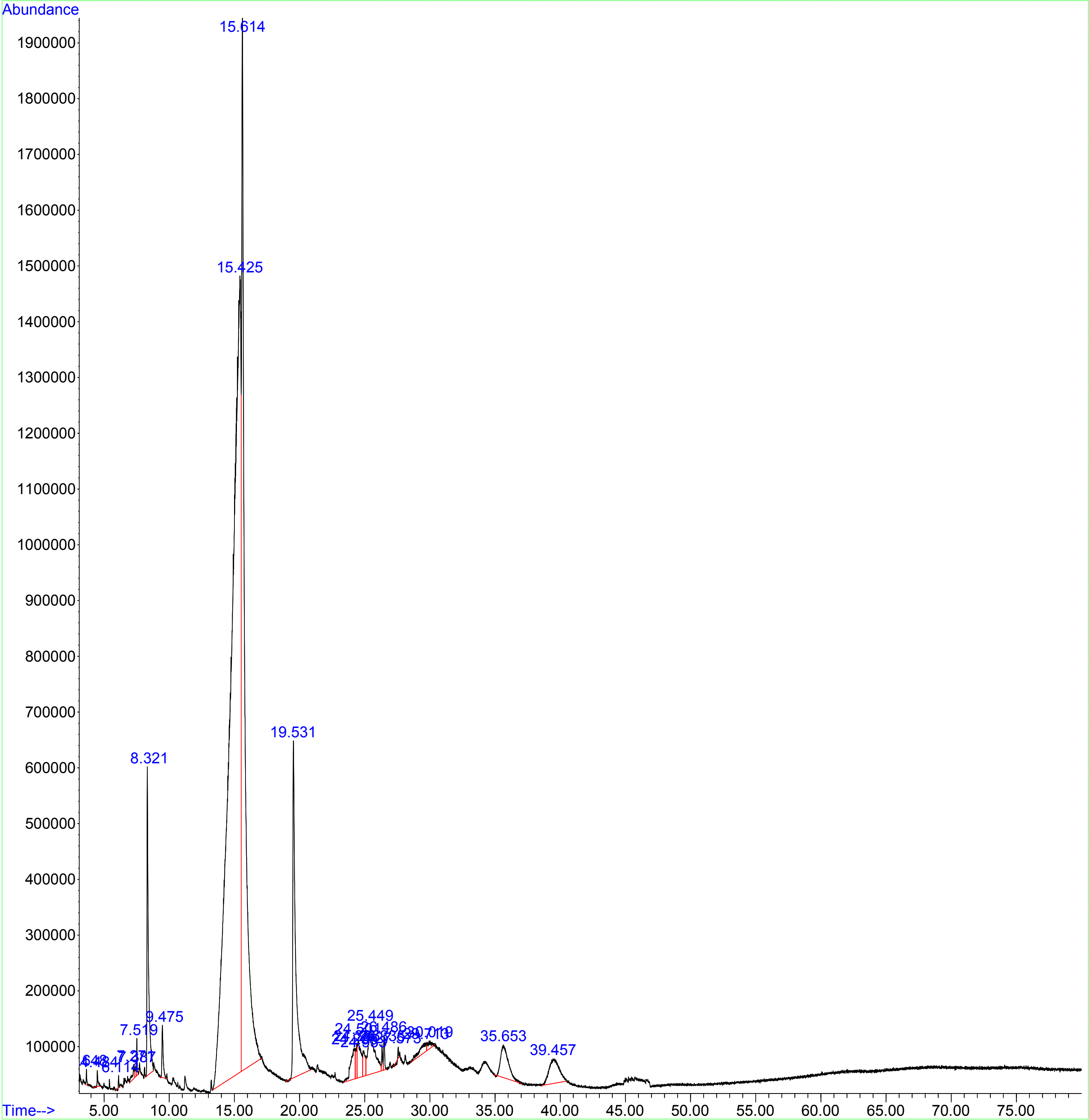

Acq On : 7 Mar 2019 14:23
Operator :
Sample : Apiary C 1
Misc :
ALS Vial : 15 Sample Multiplier: 1

Integration Parameters: autoint1.e
Integrator: ChemStation

peak #	R.T. min	first scan	max scan	last scan	PK TY	peak height	corr. area	corr. % max.	% of total
1	3.648	83	98	122	PV 2	27559	902642	0.12%	0.066%
2	4.484	186	244	283	BB	28154	1396042	0.19%	0.103%
3	6.114	491	529	544	BV 2	21425	540876	0.07%	0.040%
4	7.271	676	731	743	BV 7	24888	2173819	0.29%	0.160%
5	7.387	743	752	764	VV 3	20020	1053796	0.14%	0.078%
6	7.519	764	775	798	VV 3	64700	2118282	0.28%	0.156%
7	8.321	893	915	996	PV 3	553496	42969704	5.70%	3.164%
8	9.475	1086	1116	1166	BV 2	91822	6133895	0.81%	0.452%
9	15.425	1789	2156	2173	PV 2	1417245	753701166	100.00%	55.491%
10	15.614	2173	2189	2439	VV 4	1882196	327094424	43.40%	24.082%
11	19.531	2770	2874	3107	BV 2	603375	93341786	12.38%	6.872%
12	24.238	3545	3697	3702	BV 2	54287	12318513	1.63%	0.907%
13	24.386	3702	3722	3727	VV 2	60232	4493584	0.60%	0.331%
14	24.501	3727	3742	3801	VV 2	70734	13024712	1.73%	0.959%
15	24.933	3801	3818	3845	VV 2	45559	5890723	0.78%	0.434%
16	25.449	3845	3908	4050	VV 2	84722	28728971	3.81%	2.115%
17	26.335	4050	4063	4078	VV 4	44574	2227265	0.30%	0.164%
18	26.486	4078	4089	4128	VB 7	60125	3489066	0.46%	0.257%
19	27.573	4220	4279	4289	PV 7	20107	332440	0.04%	0.024%
20	29.713	4445	4653	4660	PV 7	14500	6271788	0.83%	0.462%
21	30.019	4660	4707	4763	VB 7	11866	2588137	0.34%	0.191%
22	35.653	5571	5691	5923	BV 7	58026	23784269	3.16%	1.751%
23	39.457	6206	6356	6537	BB 5	43469	23676446	3.14%	1.743%

Sum of corrected areas: 1358252344

Acq On : 7 Mar 2019 15:52
Operator :
Sample : Apiary C 2
Misc :
ALS Vial : 16 Sample Multiplier: 1

Integration Parameters: autoint1.e
Integrator: ChemStation

peak #	R.T. min	first scan	max scan	last scan	PK TY	peak height	corr. area	corr. % max.	% of total
1	3.645	80	98	117	BV 2	44996	1180291	0.11%	0.064%
2	8.323	888	915	1091	VB	799207	67551550	6.24%	3.670%
3	9.475	1094	1116	1196	BB 2	110712	8153984	0.75%	0.443%
4	11.218	1396	1421	1498	BB 2	44208	4171406	0.39%	0.227%
5	13.222	1732	1771	1790	BV 3	56663	2245204	0.21%	0.122%
6	15.396	1790	2151	2173	VV 3	2031808	1082215712	100.00%	58.790%
7	15.624	2173	2191	2436	VV 2	3036232	461174464	42.61%	25.053%
8	19.532	2814	2874	2997	BV 3	766432	102798811	9.50%	5.584%
9	20.324	2997	3012	3097	VB 6	42815	8049835	0.74%	0.437%
10	24.309	3549	3709	3722	BV 5	31597	7988074	0.74%	0.434%
11	24.504	3722	3743	3780	VB 5	31605	3517605	0.33%	0.191%
12	25.430	3830	3905	3965	BV 5	50475	7752139	0.72%	0.421%
13	26.485	4075	4089	4125	VB 10	44700	2288667	0.21%	0.124%
14	27.566	4127	4278	4289	BV 10	42065	8335395	0.77%	0.453%
15	29.310	4412	4583	4760	VB 10	27108	16030898	1.48%	0.871%
16	35.628	5597	5687	5930	BB 10	70357	29735272	2.75%	1.615%
17	39.370	6210	6341	6613	BB 6	47368	27616604	2.55%	1.500%

Sum of corrected areas: 1840805914

File
Operator :
Acquired : 7 Mar 2019 15:52 using AcqMethod FOMETHOD.M
Instrument : 5975 MSD
Sample Name: Apiary C 2
Misc Info :
Vial Number: 16

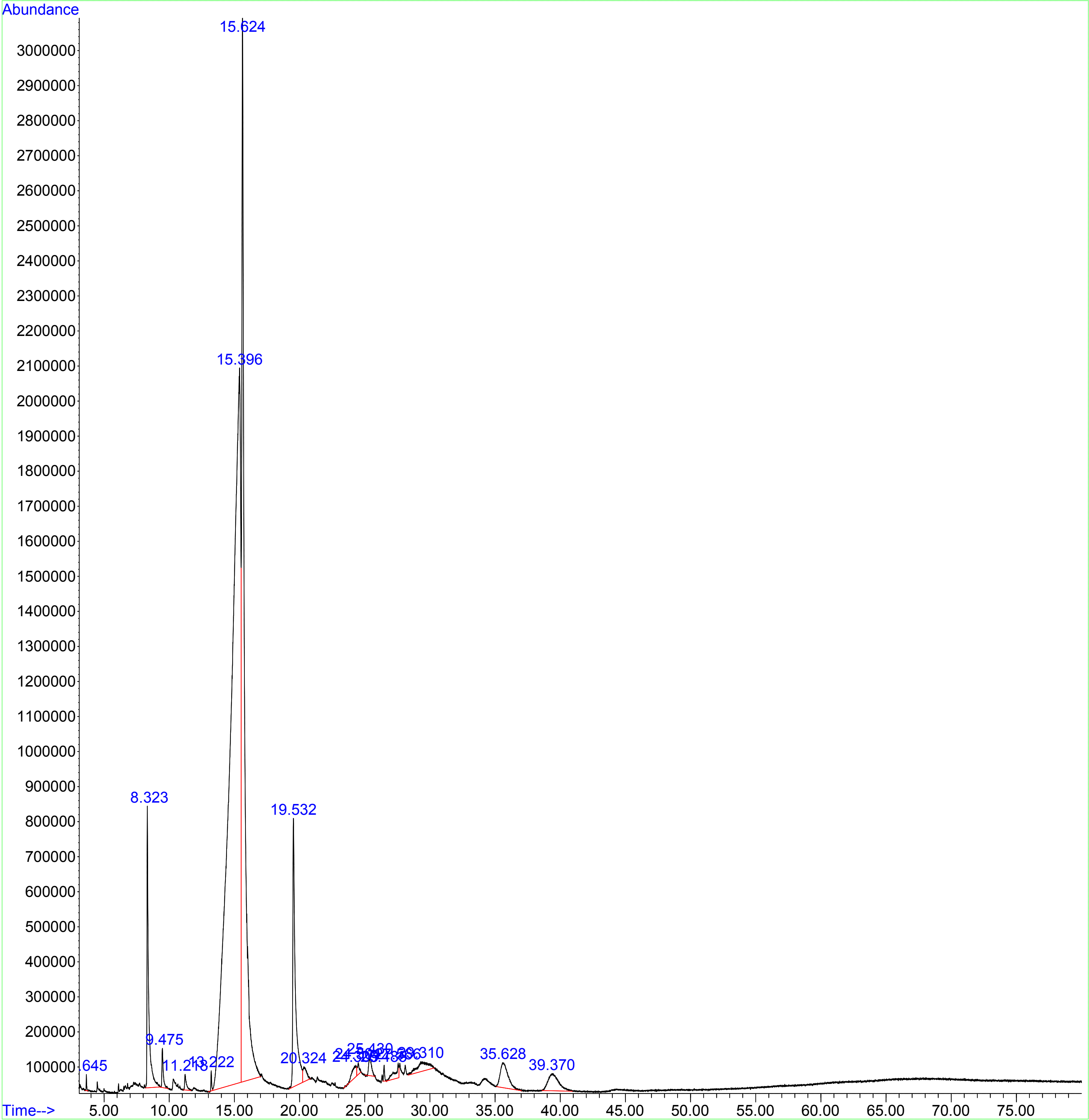

Acq On : 7 Mar 2019 17:22
Operator :
Sample : Apiary C 19
Misc :
ALS Vial : 17 Sample Multiplier: 1

Integration Parameters: autoint1.e
Integrator: ChemStation

peak #	R.T. min	first scan	max scan	last scan	PK TY	peak height	corr. area	corr. % max.	% of total
1	4.481	228	244	305	PB 3	26007	1562722	0.44%	0.182%
2	7.268	717	731	779	VB 6	28466	3185993	0.91%	0.371%
3	8.323	888	915	1034	PV	349396	27750596	7.89%	3.233%
4	9.476	1083	1117	1166	BV 2	66269	4759807	1.35%	0.555%
5	11.221	1388	1421	1499	BB	46681	4506250	1.28%	0.525%
6	14.348	1789	1968	1972	VV 4	589402	142894920	40.64%	16.650%
7	14.757	1972	2040	2164	VV 3	996688	351638203	100.00%	40.972%
8	15.611	2164	2189	2439	VV 2	1215750	170619398	48.52%	19.880%
9	19.528	2835	2873	3000	BV 2	393907	57464025	16.34%	6.696%
10	20.334	3000	3014	3055	VB 9	15503	1978689	0.56%	0.231%
11	24.510	3606	3744	3815	PB 9	29883	7961246	2.26%	0.928%
12	25.436	3832	3906	3958	BV 9	26333	4112033	1.17%	0.479%
13	26.341	4048	4064	4078	PV 6	42226	1997243	0.57%	0.233%
14	26.492	4078	4090	4122	VB 4	40082	2217135	0.63%	0.258%
15	27.573	4228	4279	4287	VV 4	23465	2539686	0.72%	0.296%
16	27.700	4287	4302	4357	VV 4	40656	4681992	1.33%	0.546%
17	28.116	4357	4374	4420	VV 4	30398	2412473	0.69%	0.281%
18	34.392	5338	5471	5594	BB 4	27867	11520686	3.28%	1.342%
19	35.767	5598	5711	5961	BB 4	89031	40607631	11.55%	4.732%
20	39.545	6226	6372	6542	BB 8	25869	13827127	3.93%	1.611%

Sum of corrected areas: 858237856

File
Operator :
Acquired : 7 Mar 2019 17:22 using AcqMethod FOMETHOD.M
Instrument : 5975 MSD
Sample Name: Apiary C 19
Misc Info :
Vial Number: 17

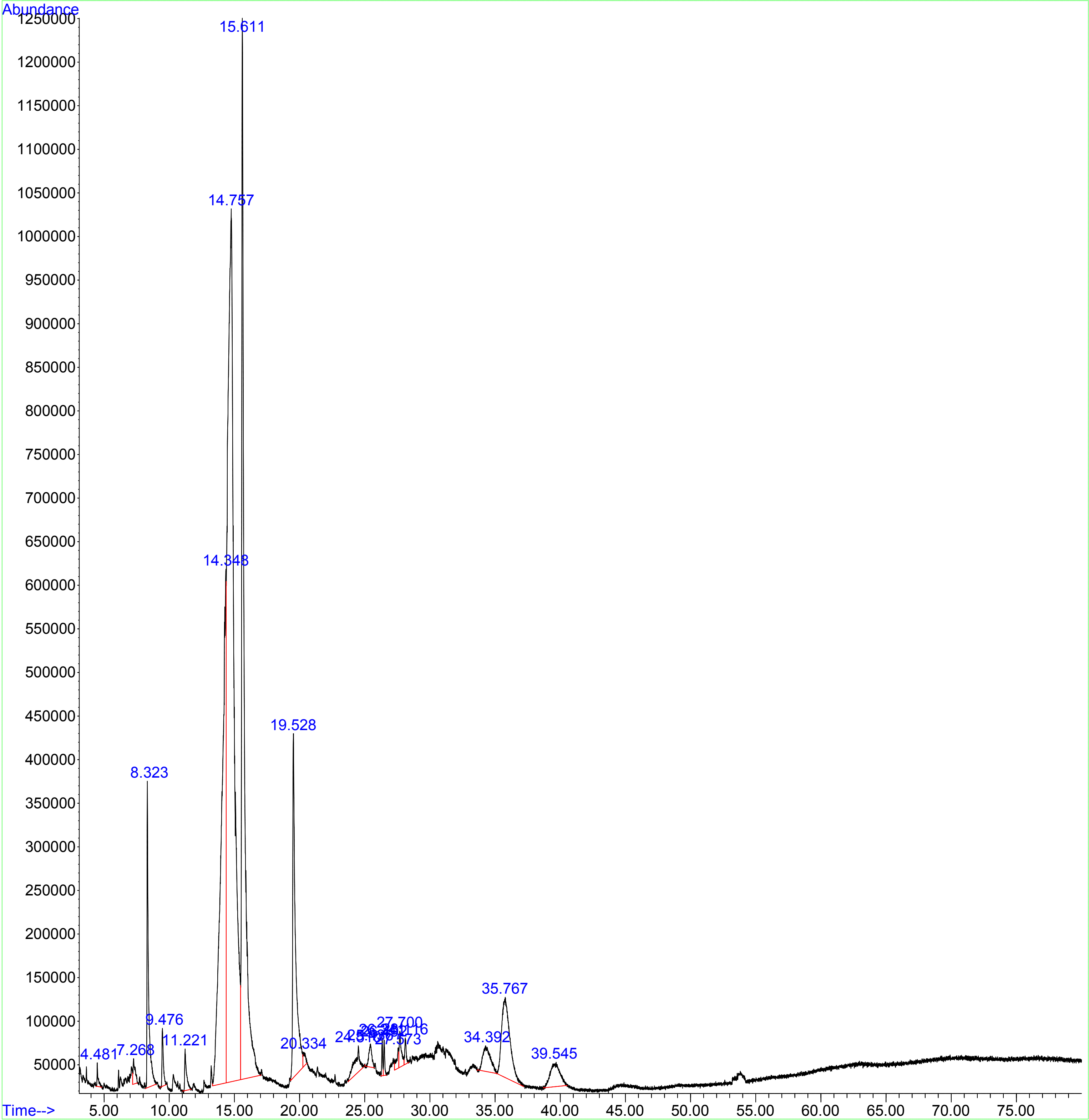

Acq On : 7 Mar 2019 18:51
Operator :
Sample : Apiary C 21
Misc :
ALS Vial : 18 Sample Multiplier: 1

Integration Parameters: autoint1.e
Integrator: ChemStation

Method
Title

peak #	R.T. min	first scan	max scan	last scan	PK TY	peak height	corr. area	corr. % max.	% of total
1	4.488	206	245	316	BB 2	30245	1951219	0.69%	0.301%
2	6.119	496	530	545	BV 2	17759	389037	0.14%	0.060%
3	6.811	627	651	673	VV 2	11409	896470	0.32%	0.138%
4	7.280	673	733	746	VV 6	23228	2313419	0.82%	0.357%
5	7.400	746	754	765	VV 5	16291	803101	0.29%	0.124%
6	7.519	765	775	798	VV 3	28304	1036332	0.37%	0.160%
7	8.331	890	917	1093	PB 2	401607	31133832	11.09%	4.803%
8	9.482	1098	1118	1165	BV 3	61107	4430561	1.58%	0.684%
9	11.226	1384	1423	1498	BB 4	36807	3077455	1.10%	0.475%
10	13.230	1734	1773	1792	BV 4	47354	2192348	0.78%	0.338%
11	14.118	1792	1928	1936	VV 2	336759	62051536	22.10%	9.573%
12	14.734	1936	2036	2165	VV 3	684187	280792501	100.00%	43.318%
13	15.620	2165	2190	2434	VV 3	844767	121228357	43.17%	18.702%
14	19.535	2830	2874	3144	PV 3	487627	85930818	30.60%	13.257%
15	24.519	3619	3746	3808	BV 8	33420	5187244	1.85%	0.800%
16	25.449	3808	3908	3955	VV 8	27702	4803472	1.71%	0.741%
17	26.346	4052	4065	4078	PV 7	47071	2186988	0.78%	0.337%
18	26.494	4078	4091	4131	VV 5	65987	3543465	1.26%	0.547%
19	27.713	4255	4304	4357	VV 5	45628	6908504	2.46%	1.066%
20	28.131	4357	4377	4423	VV 5	36078	2950830	1.05%	0.455%
21	34.428	5370	5477	5569	BB 5	18187	6089138	2.17%	0.939%
22	35.850	5591	5726	5933	BB 5	40777	18311139	6.52%	2.825%

Sum of corrected areas: 648207764

File
Operator :
Acquired : 7 Mar 2019 18:51 using AcqMethod FOMETHOD.M
Instrument : 5975 MSD
Sample Name: Apiary C 21
Misc Info :
Vial Number: 18

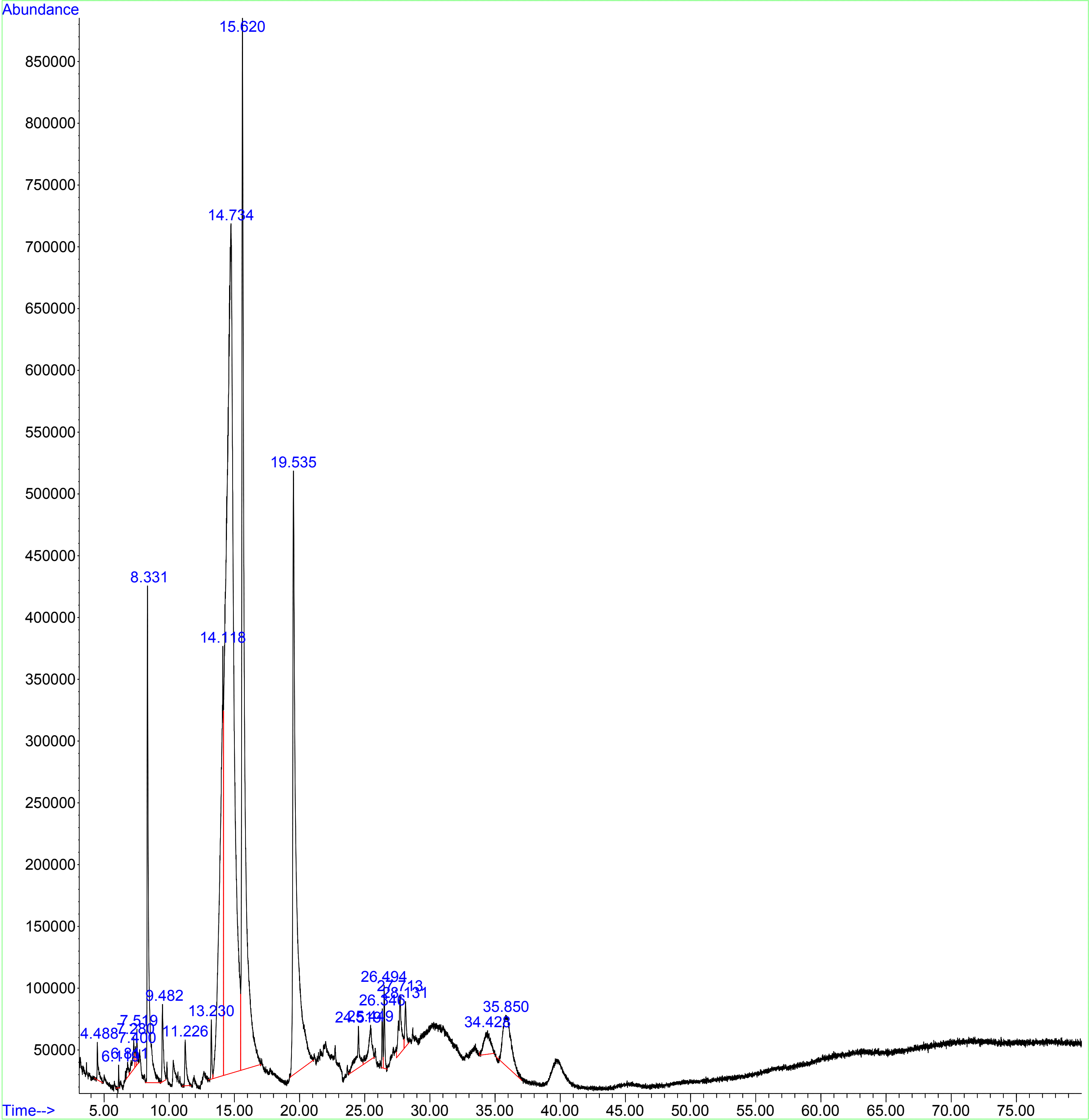

File
Operator :
Acquired : 7 Mar 2019 20:21 using AcqMethod FOMETHOD.M
Instrument : 5975 MSD
Sample Name: Apiary C 22
Misc Info :
Vial Number: 19

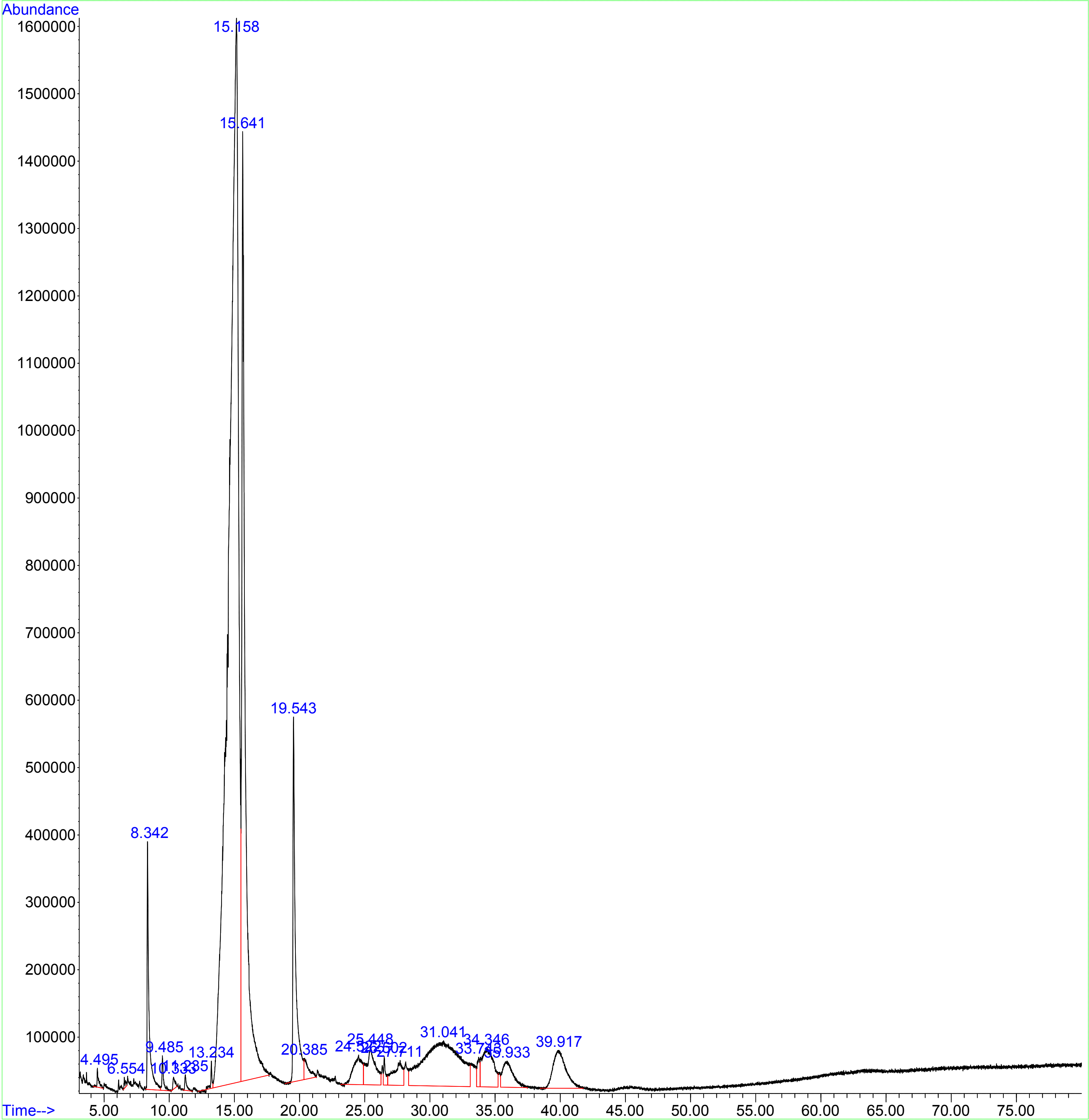

Acq On : 7 Mar 2019 20:21
Operator :
Sample : Apiary C 22
Misc :
ALS Vial : 19 Sample Multiplier: 1

Integration Parameters: autoint1.e
Integrator: ChemStation

peak #	R.T. min	first scan	max scan	last scan	PK TY	peak height	corr. area	corr. % max.	% of total
1	4.495	192	246	323	BV 5	26632	2309006	0.28%	0.153%
2	6.554	591	606	641	PV 4	15680	1027132	0.13%	0.068%
3	8.342	890	918	1092	PV 3	355953	35130724	4.29%	2.326%
4	9.485	1092	1118	1235	VB 2	48287	4363029	0.53%	0.289%
5	10.333	1235	1266	1315	BV 7	16444	1858179	0.23%	0.123%
6	11.235	1392	1424	1518	BV 2	21966	2180882	0.27%	0.144%
7	13.234	1595	1773	1794	BV 3	36675	2111031	0.26%	0.140%
8	15.158	1794	2110	2169	VV 3	1574374	819769776	100.00%	54.268%
9	15.641	2169	2194	2545	VV 3	1386256	264520518	32.27%	17.511%
10	19.543	2761	2876	3015	BV 3	538269	73996702	9.03%	4.899%
11	20.385	3015	3023	3181	VV 3	30853	7563136	0.92%	0.501%
12	24.522	3511	3746	3813	BV 3	41989	19533312	2.38%	1.293%
13	25.448	3813	3908	4048	VV 3	52914	25806212	3.15%	1.708%
14	26.502	4078	4092	4137	VV 3	41128	4425684	0.54%	0.293%
15	27.711	4137	4303	4354	VV 3	35985	16787258	2.05%	1.111%
16	31.041	4418	4885	5244	VV 9	65699	128274795	15.65%	8.492%
17	33.743	5329	5358	5377	VV 9	42857	6146355	0.75%	0.407%
18	34.346	5377	5463	5617	VV 9	55302	34703424	4.23%	2.297%
19	35.933	5650	5740	6010	VB 9	37201	21649811	2.64%	1.433%
20	39.917	6183	6437	6760	BV 6	55643	38438005	4.69%	2.545%

Sum of corrected areas: 1510594970
