# Supplementary material for: Land Use Influences the Composition and Antimicrobial Effects of Propolis
Source: Insects. 2022 Feb 28;13(3):239. doi: 10.3390/insects13030239 (PMC8950720; doi:10.3390/insects13030239)
Supplement: Supplementary file 1 [file insects-13-00239-s001.zip › insects-1593358-supplementary-Table S1,Script S1.pdf]

Table S1. Results of pairwise comparisons between apiaries with respect to propolis composition. Analyses were performed subsequent to a significant permutational ANOVA in '*RRPP*' using the '*pairwise*' function.

Comparison	Distance between Means	Z-Score	*Pr* > d
A:B	12.00	−0.03	0.502
**A:C**	**25.74**	**1.71**	**0.044**
**AD**	**26.43**	**1.86**	**0.031**
A:E	22.09	1.27	0.107
**B:C**	**27.91**	**2.10**	**0.016**
B:D	18.14	1.12	0.146
B:E	11.29	−0.18	0.557
**C:D**	**40.75**	**3.16**	**0.001**
**C:E**	**32.98**	**2.34**	**0.006**
D:E	14.70	0.54	0.301

The bolded text represents only those that were statistically significant.

Script S1

Supplementary R script of analyses from this study

```
library(RRPP)

library(ape)

library(vegan)


##Sum abundance vs cropland

PropSum<-read.csv('PropSumAbundance.csv')

summary(lm(PropSum$Mean_peak_sum_abundance~PropSum$Cropland_co
  verage))


##GCMS comparisons among apiaries

PropGCMS<-read.csv('PropGCMS.csv')

apiary<-PropGCMS[,2]

compounds<-PropGCMS[,-(1:2)]


#####Euclidean

pca.Euc<-prcomp(compounds)$x

summary(prcomp(compounds))

summary(lm.rrpp(pca.Euc~ apiary))

pair<-pairwise(lm.rrpp(pca.Euc~ apiary),groups=apiary)

summary(pair,test.type='dist')
```

```
#####Jaccard

distJac<-vegdist(compounds,method='jaccard')

pcoa.Jac<-pcoa(distJac)$vectors

summary(lm.rrpp(pcoa.Jac~ apiary))


##MIC50

PropMIC50_bigmodel<-read.csv('PropMIC50_bigmodel.csv')

summary(aov(PropMIC50_bigmodel$MIC50~PropMIC50_bigmodel$pathogen*PropMIC50_bigmodel $apiary))

PropMIC50_bigmodel$pathogen<-factor(PropMIC50_bigmodel$pathogen,levels=c('Serratia_marcescens','Klebsiella_pneumoniae','Lysinibacillus_sphaericus','Paenibacillus_larvae'))

TukeyHSD(aov(PropMIC50_bigmodel$MIC50~ PropMIC50_bigmodel$pathogen))


PropMIC50<-read.csv('PropMIC50.csv')cfa

PropMIC50_noF<-subset(PropMIC50, PropMIC50$apiary!='F')


summary(lm(PropMIC50_noF$Klebsiella_pneumoniae~ PropMIC50_noF $apiary))

TukeyHSD(aov(PropMIC50_noF$Klebsiella_pneumoniae~ PropMIC50_noF $apiary))

summary(lm(PropMIC50_noF$Serratia_marcescens~ PropMIC50_noF $apiary))

TukeyHSD(aov(PropMIC50_noF$Serratia_marcescens~ PropMIC50_noF $apiary))
```
